# Trabecular Architecture of the Proximal Tibia in Extant Hominids

**DOI:** 10.1002/ajpa.70084

**Published:** 2025-06-30

**Authors:** Andrea Lukova, Sebastian Bachmann, Alexander Synek, Dieter H. Pahr, Brandon Kilbourne, Christopher J. Dunmore, Tracy L. Kivell, Matthew M. Skinner

**Affiliations:** ^1^ Department of Human Origins Max Planck Institute for Evolutionary Anthropology Leipzig Germany; ^2^ Institute of Lightweight Design and Structural Biomechanics Wien Austria; ^3^ Leibniz Institute for Research on Evolution and Biodiversity Berlin Germany; ^4^ School of Biosciences, University of Kent Canterbury UK

**Keywords:** bipedalism, functional morphology, *Gorilla*, human, knee, locomotor behavior, *Pan*, *Pongo*

## Abstract

**Objectives:**

Extant humans and non‐human apes are characterized by diverse locomotor and postural behaviors, resulting in different joint loading patterns. These behaviors influence trabecular bone, which responds to mechanical loading and joint posture. While prior studies have examined femoral trabecular structure, this study is the first to assess trabecular architecture in the proximal tibia across extant hominoids to evaluate how differences in joint use and posture are reflected in tibial morphology.

**Materials and Methods:**

We analyze trabecular structure in the proximal tibiae of *Homo sapiens* (*n* = 25), *Gorilla* (*n* = 13), *Pan troglodytes* (*n* = 15) and *Pongo* sp. (*n* = 7). Each tibia was scanned using high‐resolution microtomography, and cortical and trabecular bone were isolated from each other. Canonical holistic morphometric analysis was used to quantify trabecular bone volume fraction and degree of anisotropy. Spatial distributions of these variables were compared across taxa using principal component analysis, and group differences were assessed with multivariate analysis of variance and pairwise tests.

**Results:**

Results show that trabecular bone volume and anisotropy reflect habitual knee posture: extended in bipedal *Homo*, and flexed in non‐human apes. In *Gorilla*, males exhibit more extended knee use than females, while no significant sex differences were observed in *Homo* or *Pan* (sex differences in *Pongo* were not tested due to sample limitations).

**Discussion:**

We demonstrate that the trabecular structure of the proximal tibia is consistent with habitual locomotor loading in extant hominids, which provides the comparative context to interpret knee posture, biomechanical loading, and predominant locomotor behaviors in fossil hominids.

## Graphical abstract

Relative bone volume (rBV/TV) mean models of the tibial plateau and tibial condyles in humans, gorillas, chimpanzees, and orangutans. Horizontal black lines through the superior view mean models show where the cross‐sectional coronal planes are positioned. Hollow black circles indicate section plane position. Red color shows the highest rBV/TV magnitude, and blue color shows the lowest rBV/TV magnitude. Trabecular bone structure across the tibial plateau and condyles varies among extant humans and non‐human apes, reflecting differences in knee loading. These differences in trabecular patterns are shaped by species‐specific knee postures and locomotor behaviors.

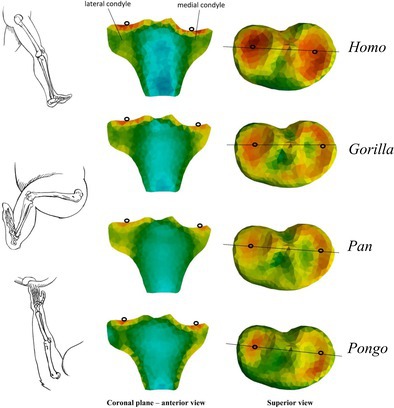

## 1 Introduction

Humans and non‐human apes are characterized by a wide range of locomotor behaviors and as such are often used as models to help reconstruct behavior in fossil hominoid taxa (Cazenave and Kivell 2023; Skinner et al. 2015; Tsegai et al. 2013; Williams et al. 2014). Particularly, the morphology of the hindlimb has played a central role for understanding the emergence of multiple forms of hominin bipedality (Berillon et al. 2010; Carey and Crompton 2005; Georgiou et al. 2020; Lovejoy and McCollum 2010; Sockol et al. 2007; Stern and Susman 1983; Susman et al. 1984). Studying the morphology of the knee specifically, including how this joint is loaded during different types of locomotion in extant apes, can provide information about how early hominins walked bipedally, as well as other potential locomotor behaviors in which they may have engaged (Carey and Crompton 2005; Lovejoy and McCollum 2010; Stern and Susman 1983; Susman et al. 1984). Previous studies have explored associations between extant hominid lower limb morphology and locomotor mode, such as Carlson (2005), who found more circular femoral cross sections in apes that engage more in arboreal locomotion, and Ruff (2002) reporting relatively stronger forelimb shafts in more arboreal species compared to more terrestrial ones. Moreover, internal bone structure differences between humans and non‐human apes, that most likely reflect differences in bone biomechanical loading during different locomotion repertoires, have been found in the hip (Cazenave et al. 2019; Cazenave et al. 2022; Chirchir 2016; Coelho et al. 2009; Dalstra et al. 1993; Demes et al. 2000; Georgiou et al. 2020; Lovejoy 1988; Ohman et al. 1997; Rafferty 1998; Saers et al. 2016; Volpato et al. 2008; Zaharie and Phillips 2018), knee (Georgiou et al. 2018; Kamibayashi et al. 1995; Lukova et al. 2024a; Mazurier et al. 2010; Novitskaya et al. 2014; Saers et al. 2016; Sylvester and Terhune 2017; Thomsen et al. 2005), and in the ankle (Barak et al. 2013; Chirchir 2016; DeSilva 2009; DeSilva and Devlin 2012; Saers et al. 2016; Sorrentino et al. 2021; Su et al. 2013; Sylvester and Terhune 2017; Tsegai et al. 2017).

Functional interpretations of external bone morphology can be complicated by phylogenetic inertia, in which some morphological traits may be due more to shared ancestry among closely related species than to the result of biomechanical demand (Briggs 2017; Cubo et al. 2008; Griffin et al. 2019; Rickman et al. 2023; Ward 2002; Wund 2012). Internal bone structure can also be influenced by genetic factors or phylogeny (Cubo et al. 2005; Lieberman 1997); however, experimental studies have previously demonstrated that cortical and, in particular, trabecular bone are able to functionally adapt their structure to the magnitude and direction of joint load (Barak et al. 2008; Barak et al. 2011; Currey 2002; Pontzer et al. 2006). Additionally, previous studies have shown an association between trabecular bone (re)modeling and the gait changes that occur with the development of bipedalism in humans (Barak 2019; Gosman and Ketcham 2009; Milovanovic et al. 2017; Raichlen et al. 2015; Ryan and Krovitz 2006). Thus, both trabecular and cortical bone can be informative for reconstructing locomotor behavior during life (Chirchir et al. 2017b; Dunmore et al. 2024; Georgiou et al. 2018; Georgiou et al. 2019; Raichlen et al. 2015; Ryan and Shaw 2012; Ryan and Van Rietbergen 2005; Saers et al. 2022; Skinner et al. 2015; Tsegai et al. 2018).

For example, in an experimental study in which mice moved within “linear” vs. “turning” tracks, Carlson et al. (2008) found limited differences in trabecular parameters in the distal femur. They attributed this result to knee joint specialization for flexion/extension in mice that may have inherently constrained mediolateral movements and reduced the impact of rapid turning maneuvers on trabecular structure. Interestingly, in a similar experimental study, Wallace et al. (2013) found differences in the trabecular structure of the mouse proximal humerus related to enhanced mediolateral and anteroposterior movements, suggesting that trabecular bone responds more in joints with greater range of motion. These findings underscore the importance of considering species with different locomotor regimes, and that the range of joint motion may play a critical role in (re)modeling trabecular architecture. In particular, variation in loading in the anteroposterior plane is reflected in the trabecular structure of sheep distal tibia in response altered ankle flexion (Barak et al. 2011) and in the sheep distal femur in response altered knee flexion (Polk et al. 2008).

Differences in range of motion can lead to varied biomechanical loading patterns across the joint surface, which, in turn, can result in a more complex and heterogenous trabecular structure as the bone functionally adapts to the different stresses it encounters. For example, Ryan and Shaw (2012) demonstrated that the variation in knee flexion and extension among humans and non‐human apes is reflected in the trabecular architecture of the distal femur. They showed that species which habitually engage in more extended postures tend to have trabecular structure aligned with the primary axis of joint loading, suggesting functional adaptation to a more consistent pattern of force application in the anteroposterior plane. Their study suggests that in species with more habitually extended limb postures, such as bipedal humans, trabecular bone may reflect more consistent joint loading patterns, leading to a more uniform trabecular architecture. Conversely, trabecular bone is likely to be more variable in its density and structural characteristics in species with more varied knee loading in flexion–extension as they navigate complex terrestrial and arboreal environments.

### 1.1 Whole‐Epiphysis Approach in Trabecular Bone Analysis

Previous studies on humans and non‐human apes have typically focused exclusively on a single epiphysis (e.g., distal femur) or region of an epiphysis (e.g., medial condyle) within the lower limb and have not consistently detected a clear postural or locomotor signal (Barak et al. 2011; Carlson et al. 2008; Ryan and Walker 2010; Shaw and Ryan 2012; Wallace et al. 2013). Several studies have demonstrated that analyzing a single subvolume of trabecular bone within an epiphysis (and relatively distant from the articular surface) tends not to identify trabecular patterning clearly related to habitual joint posture (Fajardo and Müller 2001; Kivell et al. 2011; Skinner et al. 2015; Stephens et al. 2016; Sylvester and Terhune 2017; Tsegai et al. 2013; Tsegai et al. 2018). Conversely, a whole‐epiphysis approach, which has been facilitated by recent developments in analytical software (e.g., Gross et al. 2014), can offer a more comprehensive understanding of how trabecular structure is modeled to reflect postural and locomotor joint loading.

### 1.2 Knee Posture and Loading Across Humans and Non‐Human Apes

**FIGURE 1:**
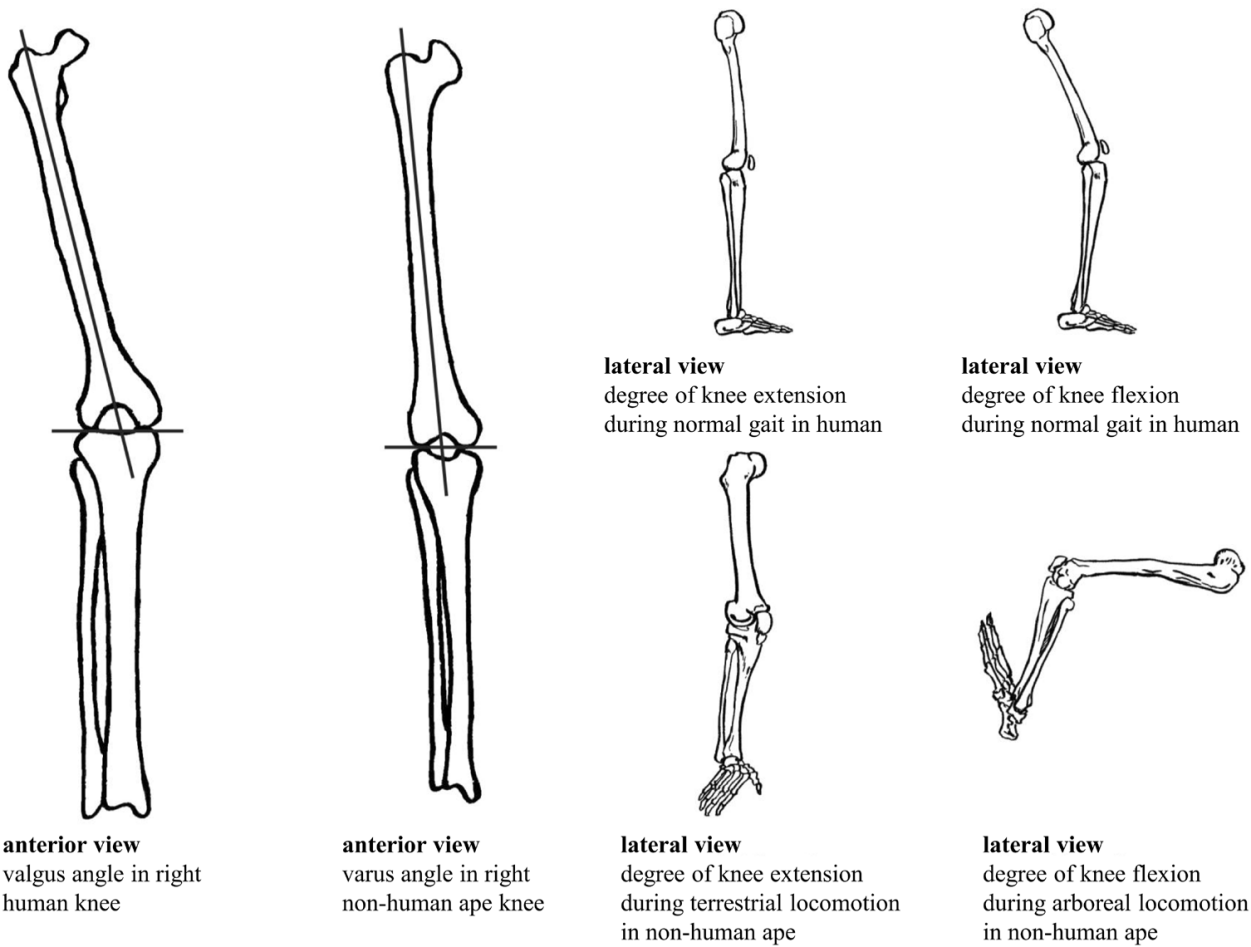
Knee angles and postures in humans and non‐human apes in anterior and lateral view. Lines in the anterior views represent the anatomical axis of the femur.

Differences in joint kinematics and frequency of specific types of locomotion, as well as variation in knee joint morphology across humans and non‐human apes (Figure 1), may influence load distribution across the tibial epiphysis and, in turn, affect trabecular bone (re)modeling (Barak 2019). Thus, understanding how humans and non‐human apes move and how they use their limbs during locomotion is essential for the interpretation of trabecular structure. Modern humans ( *Homo sapiens* ) are characterized by obligate bipedal locomotion where the knee is at, or close to, full extension during most of the walking cycle (Javois et al. 2009; Landis and Karnick 2006; Lovejoy 2007; Organ and Ward 2006; Sylvester 2013; Sylvester and Pfisterer 2012; Tardieu 1999). However, humans also often engage in other activities such as running, jumping, and squatting where the degree of knee flexion–extension may vary (Mann and Hagy 1980; Nilsson and Thorstensson 1989; Racic et al. 2009). Both the medial and lateral tibial condyles are relatively evenly loaded through the tibial plateau during human locomotion compared to non‐human apes. This even joint loading is due to hard and soft tissue adaptations of the human knee for bipedalism, which emphasizes stability in upright posture and efficient energy transfer during gait (e.g., DeSilva 2009; Richmond and Strait 2000). However, multiple studies have demonstrated that the medial compartment of the tibial plateau still experiences higher loads compared to the lateral compartment during various activities, with the highest medial compartment loading occurring during activities such as stair climbing (e.g., Andriacchi and Dyrby 2005; Heinlein et al. 2009; Kutzner et al. 2010; Liikavainio et al. 2007). The individual postural and locomotor activities habitually practiced by our human skeletal sample are unknown. Therefore, we assume that all sampled individuals primarily loaded their tibia through bipedal walking but acknowledge that they likely engaged in other activities that may be reflected in the trabecular structure of the proximal tibia as well.

*Gorilla* and *Pan* both engage most frequently in quadrupedal knuckle‐walking but also engage in other terrestrial and arboreal locomotor activities [Bauer 1977 (wild *Pan*); Doran 1993 (wild *Pan*); Doran 1997 (wild *Gorilla* and *Pan*); Drummond‐Clarke et al. 2022 (wild *Pan*); Hunt 1992 (wild *Pan*); Isler 2005 (captive *Gorilla* and *Pan*); Remis 1994 (wild *Gorilla*); Tocheri et al. 2011 (wild *Gorilla*)]. Their knee is typically flexed to varying degrees during both terrestrial and arboreal locomotion [D'Août et al. 2004 (wild *Pan*); Finestone et al. 2018 (captive *Gorilla* and *Pan*); Georgiou et al. 2018 (wild *Gorilla* and *Pan*); Isler 2005 (captive *Gorilla* and *Pan*); Pontzer et al. 2009 (captive *Pan*)]. In both *Gorilla* and *Pan*, the knee is mostly loaded in flexed and varus postures where the medial tibial condyle is loaded more than the lateral tibial condyle during both arboreal and terrestrial locomotion [Watson et al. 2009 (captive *Gorilla* and *Pan*); Tardieu 1981 (captive *Gorilla* and *Pan*)]. The differing size between femoral condyles in *Gorilla* and *Pan* causes mediolateral knee rotation [Freeman and Pinskerova 2005 (captive *Gorilla* and *Pan*); O'Neill et al. 2013 (wild *Pan*)] during all phases of terrestrial quadrupedal locomotion [Sylvester 2013 (wild *Gorilla* and *Pan*); Tardieu 1999 (captive *Gorilla* and *Pan*); Sylvester and Pfisterer 2012 (wild *Gorilla* and *Pan*)]. Previous studies have found that *Gorilla* extends their knees more during both terrestrial and arboreal locomotion (particularly vertical climbing) compared to *Pan* [Finestone et al. 2018 (captive *Gorilla* and *Pan*); Isler 2005 (captive *Gorilla* and *Pan*); Kozma et al. 2018 (wild *Gorilla* and *Pan*)]. In our study, *Gorilla* is represented only by wild *Gorilla gorilla gorilla*, in which females are more arboreal and show a greater range of motion at the hip and knee [Hammond 2014 (captive *Gorilla* and *Pan*); Isler 2005 (captive *Gorilla* and *Pan*)]. Additionally, a recent study by King et al. (2025) has shown that male and female wild western lowland *Gorilla* engage in different arboreal locomotions. They found that particularly male silverbacks engage in less canopy locomotion, stay closer to the ground, and use larger supports compared to females, who may adjust their arboreal behavior based on their reproductive status to manage risk during gap‐crossing (King et al. 2025). In *Pan*, the knee is mostly loaded in a flexed and varus posture, though lateral rotation of the knee occurs during extension, which increases load in the lateral knee compartment (Lovejoy 2007). During climbing, *Pan* species may fully utilize the entire flexion–extension range of the knee [D'Août et al. 2002 (mixed *Pan*); Isler 2005 (captive *Gorilla* and *Pan*)]. In this study, *Pan* is represented exclusively by wild *Pan troglodytes verus* from the Taï National Forest in Ivory Coast. Previous research has found no significant sex differences in the overall frequency of arboreal and terrestrial locomotion within this community of chimpanzees [Doran 1993 (wild *Pan*)].

*Pongo* is the most arboreal of non‐human apes, engaging most frequently in torso‐orthrograde suspension, vertical climbing/descent and arboreal quadrupedal/tripedal walking [Thorpe and Crompton 2006 (wild)]. *Pongo* also uses bipedality and hindlimb suspension during arboreal locomotion in which their knee posture ranges from hyperflexed to extended [Cant,1987 (wild); Isler 2005 (captive); Manduell et al. 2012 (wild); Payne et al. 2006 (wild); Thorpe and Crompton 2006 (wild); Thorpe et al. 2009 (wild)]. During arboreal locomotion, *Pongo* shows a significantly larger flexion–extension range of motion at the knee joint, loading the proximal tibia in a greater range of postures relative to African apes [Isler 2005 (captive)]. However, during terrestrial locomotion, flexion–extension range of motion at the knee in *Pongo* does not differ significantly from that of African apes [Kozma et al. 2018 (wild)]. In this study, *Pongo* is represented by wild *Pongo pygmaeus* and wild *Pongo abelii* . In both subspecies, females have been found to be more arboreal compared to males [Cant 1987 (wild); Galdikas 1988 (wild)].

We investigate the trabecular structure in the entire proximal tibial epiphysis of extant humans and non‐human apes (*Pan*, *Gorilla* and *Pongo*) to explore how differences in knee joint loading during locomotion potentially impact trabecular structure. Only three previous studies have specifically examined trabecular bone structure within the knee joint in a sample of humans and non‐human apes (Georgiou et al. 2018; Lukova et al. 2024a; Sylvester and Terhune 2017), and all of these focused only on the distal femur. Trabecular structure in the proximal tibia has been investigated exclusively in humans (Kamibayashi et al. 1995; Novitskaya et al. 2014; Saers et al. 2016; Sugiyama et al. 2012; Thomsen et al. 2005), with the exception of Mazurier et al. (2010) who compared humans with non‐human apes; however, their focus was only on the cortico‐trabecular complex underlying the tibial plateau. We build upon the previous studies with a canonical holistic morphometric analysis (cHMA) approach to statistically analyze trabecular patterns free of a priori subsampling (Bachmann et al. 2022).

## 2 Hypotheses and Predictions

In this study, we quantify relative trabecular bone volume (relative bone volume/total volume, rBV/TV) and the degree to which trabeculae are similarly aligned (degree of anisotropy, DA) throughout the whole proximal tibia epiphysis. We also quantify absolute trabecular bone volume (bone volume/total volume, BV/TV) in the tibial plateau only. In this first application of the cHMA method to the proximal tibia, we restrict our analysis to these variables as they have been shown to explain up to 97% of the variation in elastic properties of trabecular bone in humans (Homminga et al. 2003; Maquer et al. 2015; Van Rietbergen et al. 1998; Zysset 2003). The cHMA method can be used to examine additional trabecular bone variables (e.g., trabecular thickness, spacing and number), but these will be the focus of future work. Below we outline three hypotheses based on the current literature of knee joint kinematics in humans and non‐human apes reviewed above.

### 2.1 First Hypothesis

**FIGURE 2:**
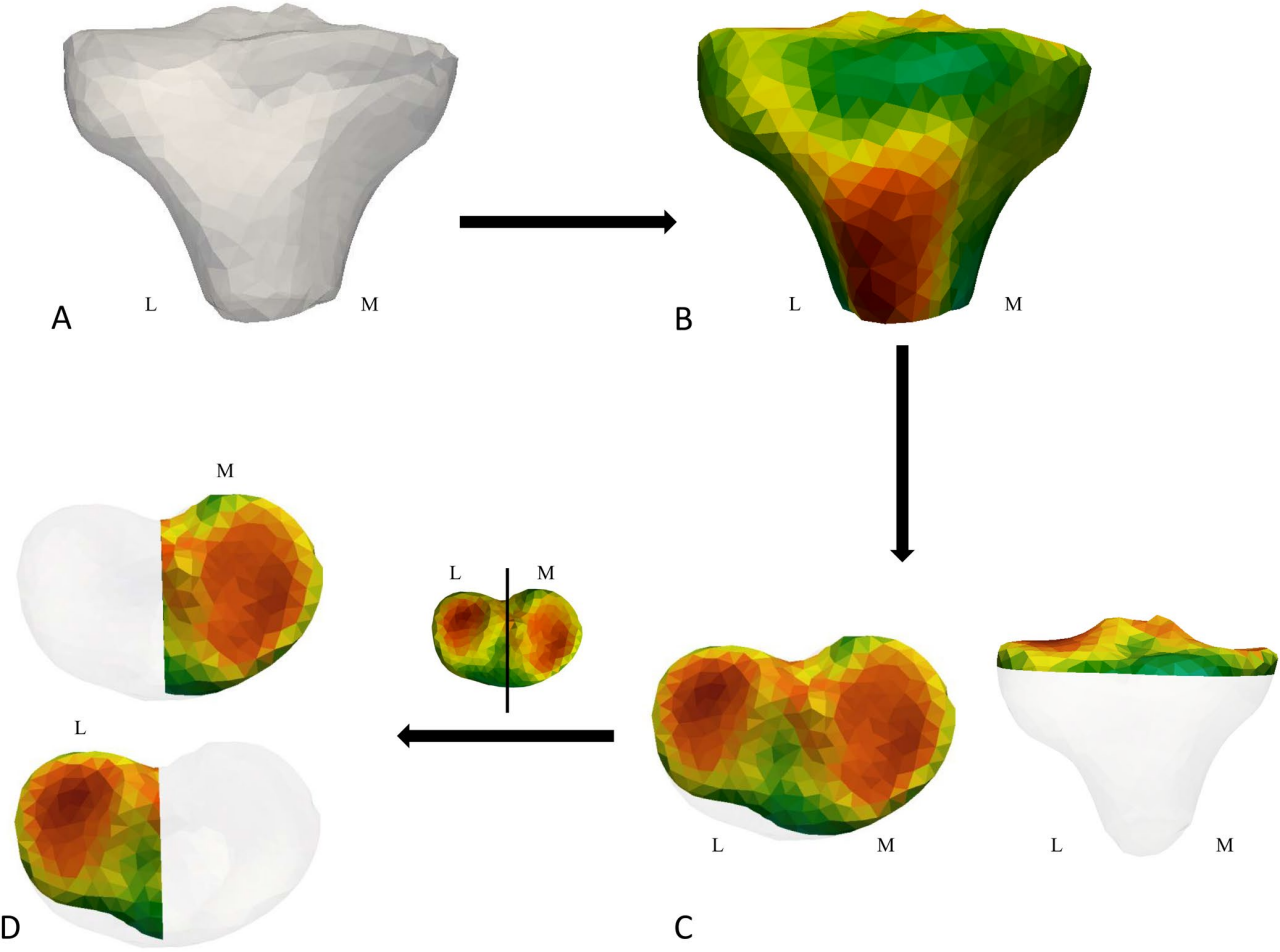
(A) Model of outer canonical atlas showing orientation and external morphology of the right proximal tibia computed by cHMA. (B) Inner mesh showing rBV/TV distribution in the whole proximal tibia of the human sample computed by cHMA. Note that the inner mesh of rBV/TV, as well as DA distribution, was generated for each taxon. (C) The cropped tibial plateau used to test for differences in mean BV/TV between taxa. (D) This tibial plateau subsection was further cropped in the sagittal plane to test for differences in mean BV/TV between the medial and lateral condyles.

Our first hypothesis is that trabecular architecture of the proximal tibia (Figure 2A,B) will reflect differences in frequently observed knee postures and presumed knee loading during locomotor behaviors across humans (*Homo*), *Pan*/*Gorilla*, and *Pongo*. We predict that:

1A) Due to similar loading of the medial and lateral tibial condyles, we predict that BV/TV distributions in each condyle will be similar in *Homo*. We predict that high BV/TV values on the tibial plateau will be concentrated in the anteroposterior center of each condyle due to an extended knee posture during most of the bipedal walking cycle (Javois et al. 2009; Landis and Karnick 2006; Lovejoy 2007; Organ and Ward 2006; Sylvester 2013; Sylvester and Pfisterer 2012; Tardieu 1999). Additionally, we predict that *Homo* will exhibit lower mean BV/TV in the proximal tibia compared to *Gorilla* and *Pan*. This is based on previous findings that show a relative reduction in trabecular bone volume in modern humans, likely related to a combination of reduced overall mechanical loading and a more stereotyped locomotor repertoire dominated by extended lower limb postures during bipedal walking (Chirchir et al. 2015, 2017a; DeMars et al. 2021; Ryan and Shaw 2015; Saers et al. 2016, Tsegai et al. 2018).

1B) *Pan* and *Gorilla* will exhibit the highest BV/TV in the medial tibial condyle due to higher medial knee compartment loading. The highest BV/TV values under the tibial plateau will be concentrated more medially on the medial condyle due to their more flexed knee position and varus knee angle (compared to *Homo*). Moreover, based on the previous studies of *Gorilla* locomotion, which have shown that *Gorilla* extends their knees more, we predict higher lateral condyle loading (i.e., higher BV/TV values in the lateral condyle) in *Gorilla* compared to *Pan* (D'Août et al. 2004; Finestone et al. 2018; Isler 2005; Pontzer et al. 2009).

1C) *Pongo* will exhibit a more homogeneous spatial distribution of BV/TV values across the proximal tibia relative to other non‐human apes due to their more variable knee postures (that vary from full extension to full flexion) and knee loading during locomotion. However, higher BV/TV in the medial condyle is still expected due to the higher medial knee compartment loading compared to *Homo* (caused by a varus knee angle) in *Pongo*. We expect differences in statistical distribution between the condyles will be lower compared to *Pan* and *Gorilla* (Isler 2005; Payne et al. 2006; Thorpe et al. 2009; Thorpe and Crompton 2006).

1D) We expect to find higher BV/TV values under the insertions of the patellar ligament (tibial tuberosity) in all studied taxa. Higher BV/TV values in the non‐human apes are expected due to the stress that the knee experiences under the patellar ligament during deep flexion (e.g., D'Août et al. 2002; Sylvester 2013). High BV/TV values in *Homo* are expected due to the substantial forces transmitted through the knee joint during activities involving both knee extension and controlled flexion. The patellar ligament plays a key role in this loading by transmitting quadriceps‐generated force to the tibial tuberosity, enabling knee extension. This mechanism is essential not only for bipedal walking but also for movements such as running, jumping, squatting, and sitting, where the knee sustains load in various flexed positions. We also expect to find high mean BV/TV values under the tibiofibular joint articulation in all taxa as any load transmitted into the distal fibula that is transferred vertically into the proximal tibial epiphysis passes through this articulation (e.g., Crompton et al. 2010; Holowka et al. 2017; Pietrobelli et al. 2023; Sarma et al. 2015).

1E) In the regions of stereotypical knee loading, the trabeculae will be oriented with a dominant principal alignment (high DA), while regions with diffuse loading will be oriented without any principal alignment (low DA). Thus, we expect DA to be the lowest on the margins of the tibial plateau and high in the center of each articular surface where the condyles predominantly articulate. We expect *Homo* to exhibit higher DA directly beneath both condyles in relation to the other taxa due to their stereotypically extended knee loading and relatively higher body mass loading during bipedal walking.

In addition to analyzing trabecular distribution throughout the entire proximal tibia, we also assess statistical differences in BV/TV values in the cropped tibial plateau region (Figure 2C,D). This more restricted region of interest allows for direct comparison with previous studies focusing specifically on the knee joint surface (e.g., Chirchir et al. 2015; Ryan and Krovitz 2006), and facilitates clearer visualization of compartment‐specific loading patterns.

### 2.2 Second Hypothesis

The second hypothesis is that trabecular structure will reflect taxon‐specific sex differences (or lack thereof) in the locomotor behaviors of *Homo*, *Pan*, and *Gorilla* (sex differences could not be tested in *Pongo* due to limited sample size; Table 1 and S1). We expect that the presence and extent of sex differences in trabecular parameters will vary according to the degree of sexual dimorphism in each taxon's locomotor or postural behavior. We predict that:

2A) Female *Gorilla* will exhibit higher BV/TV and higher DA values in the medial tibial condyle compared to males. This expectation is based on evidence that females more frequently engage in climbing, which likely results in increased loading of the medial compartment of the knee (Hammond 2014; Isler 2005).

2B) There will be no sex differences in BV/TV and DA spatial distribution in *Pan* or *Homo* related to function, since significant sex differences in the locomotor repertoire or knee posture have not been previously documented in these taxa (Doran 1993).

## 3 Materials and Methods

### 3.1 Study Sample, Scanning, and Segmentation

The study sample consists of complete proximal tibiae of *Homo sapiens* , *Gorilla gorilla gorilla* , *Pan troglodytes verus* and *Pongo* sp. All specimens were skeletally mature and exhibited no pathologies. All non‐human apes were wild born. Details of the study sample are shown in Tables 1 and S1. We acknowledge that the sample size for some taxa is low; however, a similar sample size (or smaller) has been used in many previous lower limb trabecular studies investigating locomotor repertoires in humans and non‐human apes (e.g., Cazenave et al. 2019; Georgiou et al. 2020; Mazurier et al. 2010; Su et al. 2013; Sylvester and Terhune 2017). The *Gorilla* sample is from Cameroon and curated at the Powell‐Cotton Museum in Birchington‐on‐Sea, United Kingdom. The *Pan* sample is from the Taï Forest National Park, Ivory Coast and curated at the Max Planck Institute for Evolutionary Anthropology in Leipzig, Germany. The *Pongo* sample consists of five individuals of *P. pygmaeus* , one *P. abelii* (female) and one *P.* sp. (female). Human specimens were drawn from three populations. Nine individuals (GAUG‐Inden from Germany) of our human sample originate from diverse postindustrial populations and six individuals (NGA and NGB specimens) are from the Medieval period (11th to 16th centuries AD) excavated from St Gregory's Priory in Canterbury, United Kingdom. Ten individuals (all males) derive from the Mary Rose shipwreck from early 16th century (Table 1). There are currently no known associations with living descendants/communities in our human sample. All data collection was conducted in line with the ethical guidelines of each curatorial institution (i.e., Johann‐Friedrich‐Blumenbach Institute for Zoology and Anthropology, Mary Rose Trust, and Skeletal Biology Research Centre at the University of Kent). For most of our sample (62%) we used the right proximal tibia. However, when it was not possible due to preservation the mirrored left proximal tibia was used.

**TABLE 1:** Sample composition and voxel size range.

Taxon	Prevalent locomotor behavior	N	Sex			Voxel size (mm)	
			Female	Male	Unknown	Min	Max
*Homo sapiens* (GAUG‐Inden)^a^	Bipedal	9	3	6	0	0.036	0.036
*Homo sapiens* (Mary Rose)^b^	Bipedal	10	0	10	0	0.033	0.038
*Homo sapiens* (NGA)^c^	Bipedal	5	3	2	0	0.030	0.036
*Homo sapiens* (NGB)^c^	Bipedal	1	0	0	1	0.036	0.036
*Gorilla gorilla gorilla*	Terrestrial knuckle‐walker/Arboreal	13	7	6	0	0.048	0.058
*Pan troglodytes verus*	Terrestrial knuckle‐walker/Arboreal	15	9	6	0	0.029	0.030
*Pongo* sp.	Arboreal/Ratcheting	7	5	2	0	0.027	0.030
Total		60					

^a^ University of Gottingen, Gottingen, Germany. Postindustrial population—low level of activity.

^**b**^ Mary Rose Trust, Portsmouth, United Kingdom. Mary Rose shipwreck—high level of activity.

^c^ University of Kent, Canterbury, United Kingdom. Postindustrial population—low level of activity.

Specimens were scanned using a BIR ACTIS 225/300 or Diondo D3 high‐resolution micro‐CT scanner housed at the Department of Human Evolution, Max Planck Institute for Evolutionary Anthropology (Leipzig, Germany), a Phoenix Nanotom S—X‐ray tomograph at the Department of Micro‐CT Laboratory, Museum of Natural History (Berlin, Germany), a Nikon 225/XTH scanner at the Cambridge Biotomography Centre, University of Cambridge (Cambridge, UK), or with a Diondo D1 scanner at the Imaging Centre for Life Sciences at the University of Kent (Canterbury, UK). The scan parameters included acceleration voltages of 100–160 kV and 100–140 μA using a 0.2–0.5 mm copper or brass filter. Scan resolution ranged between 0.027 and 0.058 mm, depending on the size of the bone and scanner used (Table 1; Table S1), which is consistent with the threshold for sufficient trabecular bone analysis (Lukova et al. 2024b). Images were reconstructed as 16‐bit TIFF stacks. All images were then segmented into binary phases of background and bone using the MIA‐clustering algorithm (Dunmore et al. 2018).

### 3.2 Trabecular Bone Analysis

Analysis of trabecular bone was conducted in medtool 4.5 (http://www.dr‐pahr.at/medtool/) following published protocols (Gross et al. 2014; Pahr and Zysset 2009; Tsegai et al. 2013). This process involves morphological filters to fill the bone and the use of a ray‐casting method to isolate the external and internal edge of the cortex in 3D, resulting in a mask of the internal bone volume and outer cortex. Using holistic morphometric analysis (HMA), the trabecular volume is analyzed using a rectangular background grid and overlapping spherical 5 mm volumes of interest (VOI), centered on each of the grid's 2.5 mm spaced vertices. The ratio of bone volume to total volume (BV/TV) and degree of anisotropy (DA) of each VOI is then calculated.

BV/TV is the proportion of trabecular bone within the total volume in each region. Regions with high BV/TV are assumed to reflect higher or more frequent biomechanical loading following the concept of bone functional adaptation (Pontzer et al. 2006; Ruff et al. 2006). However, absolute values of BV/TV have previously been argued to show systemic differences across extant hominids (Dunmore et al. 2024; Ryan and Shaw 2012; Saers et al. 2016; Tsegai et al. 2018) and so relative measures have often been used to test for interspecific differences in trabecular bone volume spatial distribution (e.g., Dunmore et al. 2019; Dunmore et al. 2020; Dunmore et al. 2024; Sukhdeo et al. 2020). Here, to compensate for potential systemic differences across our sample taxa and to analyze bone volume spatial distribution while controlling for the overall bone volume density, the BV/TV of each tetrahedron (see below) was divided by the overall average for that individual to give a measure of relative bone volume (rBV/TV). rBV/TV shows where bone volume is higher or lower relative to the mean, allowing for comparisons of trabecular bone volume spatial distribution between individuals and species that may differ in absolute BV/TV (Dunmore et al. 2019, 2024; Sukhdeo et al. 2020).

DA describes the degree to which trabecular struts are similarly aligned in 3D space, with high DA indicating greater similarity in alignment and low DA indicating a lack of similarity in alignment (Harrigan and Mann 1984). DA was calculated using the mean‐intercept‐length method (Odgaard et al. 1997; Whitehouse 1974). The value of DA is zero if the minor and major orientations are of equal magnitude, that is, isotropic, and is one if the minor and major orientations are of maximally different magnitudes, that is, anisotropic.

BV/TV and DA values are linearly interpolated onto a tetrahedral mesh of the trabecular volume. BV/TV and DA values generated by HMA can be measured in individual CT images and then interpolated on the morphed canonical bone mesh. The individual morphed meshes can then be morphed back to the canonical shape with individual HMA values mapped to homologous tetrahedral elements in the mesh, allowing for geometrically homologous volumetric comparisons. All color‐coded meshes were then visualized in Paraview v4.4.0 for the qualitative interpretations of the quantitative results generated by cHMA, where warm (red) colors represent high values and cool (blue) colors represent low values of rBV/TV and DA.

cHMA was used to quantitatively analyze the rBV/TV and DA values in the meshes of each proximal tibia following published protocols (Bachmann et al. 2022). In brief, this method first creates a canonical (i.e., average) proximal tibia from all the samples (all proximal tibia used in the sample), including both the outer shape as well as the internal trabecular volume (Figure 2A,B). Second, all individuals are registered onto this canonical bone to establish homology in orientation, size, and position. To create a canonical bone for all taxa, we used 15 humans, 13 gorillas, 15 chimpanzees and 7 orangutans. We did not use the complete human sample (*n* = 25) to avoid biasing the canonical bone toward a human shape of the proximal tibia and to better represent morphology of all the taxa in our sample. The 15 human specimens used to create the canonical bone model were chosen randomly across the human populations. The remaining 10 human individuals were used for all further analysis to increase statistical power. The same canonical bone mesh was used for all statistical analyses studying both interspecies and sex differences. Although the smaller sample of orangutans could potentially create a canonical tibia that is slightly less representative of *Pongo* than the other species in our sample, the external morphology of proximal tibia is similar across non‐human apes and thus we expect bias from sample size differences to be minimal.

Previous studies have indicated that the insertion points of tendons and ligaments as well as small articular facets can be identified in the underlying trabecular structure. In this study we were interested in trabecular bone architecture below the insertion of the patellar ligament and the tibiofibular joint. Insertion locations of the patellar ligament and the tibiofibular joint articulation were identified (based on their anatomical location for each studied species) on the individual tibial models, but interpreted within the mean canonical model, and are therefore referred to as the “presumed insertion” and “presumed articulation,” respectively. Trabecular concentrations (high rBV/TV values) found under all ligaments and joints of each undeformed individual bone were qualitatively checked to see if the morphed model is similar to the original individual models using the holistic morphometric analysis (HMA) of medtool 4.5.

### 3.3 Statistical Analysis

All quantitative comparisons of measured variables and statistical analyses were conducted on the data generated from cHMA as the tetrahedral elements can be considered geometrically homologous between individual bones (Bachmann et al. 2022). To analyze the spatial and statistical distribution of trabecular bone in each taxon, a principal component analysis (PCA) was run for rBV/TV and DA separately. The values of these trabecular measures at each tetrahedral element of the canonical mesh were treated as input variables for the PCA. PCA loadings representing three signed standard deviations (SD) of each principal component (PC) were mapped to the canonical mesh situated at the positive and negative ends of each axis. Using these loading models, we visualize the extreme patterns of rBV/TV (indicating regions with the highest rBV/TV values) and DA (showing regions with the highest DA values) that drive variance along the axes. Based on the rBV/TV and DA data spatial distribution across the individuals in our sample, we thresholded the resultant models to only include those rBV/TV and DA values above the 60th percentile of trabecular values. This allowed volumetric visualization of the tibia regions that most strongly drive the variation observed along each PC. Pairwise permutational MANOVAs (Table 2) were conducted on scores from the first two to four principal components, depending on the dataset. Pairwise comparisons were conducted on the PC scores, not the raw parameters, and were used to test for significant inter‐ and intrataxon (i.e., sex) differences in these reduced multivariate spaces. The number of PCs was selected based on a 10% variance‐explained threshold and visual inspection of scree plots. In each case, only those PCs that exceeded this threshold or contributed meaningfully to group separation were included. For example, when running MANOVA on rBV/TV variation among species, PC3 explained slightly less than 10% of the variance but was retained because it captured a clear separation of *Pongo* from African apes. While these selected PCs do not represent the full multivariate distribution, they summarize the major axes of variation, accounting for the most biologically meaningful patterns in the data.

**TABLE 2:** Interspecific and intraspecific pairwise permutational MANOVAs on the first three principal components of rBV/TV (green) for the differences between species, on the first four principal components of rBV/TV (green) for the sex differences in *Gorilla,* on the first two principal components of DA (blue) for the differences between species, and on the first two principal components of DA (blue) for the sex differences in *Gorilla*.

	*Homo*	*Gorilla*	*Pan*	*Pongo*		*Gorilla* F	*Gorilla* M
*Homo*		**< 0.001**	**< 0.012**	> 0.999	*Gorilla* F		**< 0.001**
*Gorilla*	**0.006**		0.999	**< 0.001**	*Gorilla* M	0.063	
*Pan*	**0.006**	0.012		0.235			
*Pongo*	0.012	**0.006**	**0.006**				

To test for significant differences in absolute BV/TV in the tibial plateau only, we cropped tibiae just below the most inferior point of the subchondral surface in Paraview v4.40 (Figure 2C). This cropping method was applied independently to each specimen, ensuring that only the tibial plateau was consistently isolated for comparison. By focusing on this region, we could directly analyze trabecular structure within the articular surface of the knee, a region frequently analyzed in previous studies (e.g., Chirchir et al. 2015; Ryan and Krovitz 2006; Saers et al. 2016). Analyzing this localized region complements the whole epiphysis by providing additional assessment of compartment‐specific joint loading patterns that may be obscured in broader analyses of the entire proximal tibia. Within this cropped region, we calculated mean BV/TV of each specimen of the tibial plateau. Additionally, we divided the tibial plateau at the intercondylar eminence (in Paraview v4.40) and calculated mean BV/TV for the medial and lateral sides separately (Figure 2D). To test for inter‐ and intrataxon differences, we run the Kruskal–Wallis and post hoc Dunn's tests with a Bonferroni correction (Supplementary Table S2; Table 3) on the averages of the medial and lateral condyles. Significance was determined at α = 0.05 for all statistical tests. All statistical tests, PCA plots and boxplots were done in R v4.2.2 using the *rgl*, *dunn.test* and *stats* packages (*R Core Team, 2017*).

**TABLE 3:** Dunn's tests for interspecific differences in mean BV/TV throughout the tibial plateau (orange), the lateral side of the tibial plateau (green), and the medial side of the tibial plateau (blue). Dunn's tests for intraspecific differences in mean BV/TV between the medial and the lateral sides of the tibial plateau (yellow).

	*Homo*	*Gorilla*	*Pan*	*Pongo*	*Homo*	*Gorilla*	*Pan*	*Pongo*		Medial vs. lateral
*Homo*						**< 0.001**	**< 0.001**	0.999	*Homo*	0.999
*Gorilla*	**< 0.001**				**< 0.003**		0.999	**0.01**	*Gorilla*	**0.001**
*Pan*	**< 0.001**	0.999			**< 0.003**	0.999		**< 0.021**	*Pan*	**0.031**
*Pongo*	0.999	**0.008**	**< 0.010**		0.999	**< 0.007**	**< 0.007**		*Pongo*	**0.001**

For clarity, we use the term “spatial distribution” to describe the spatial arrangement of rBV/TV or DA within a specific anatomical region (e.g., medial condyle), the term “concentration” to refer to high rBV/TV values when describing the spatial distribution of a given region, and the term “statistical distribution” to describe the statistical evaluation of mean differences (e.g., rBV/TV within the PCA plots or BV/TV within box plots).

## 4 Results

### 4.1 Relative Bone Volume in the Proximal Tibia

#### 4.1.1 Spatial Distribution in the Whole Proximal Tibia

The quantitative and qualitative descriptions of rBV/TV statistical (quantitative) and spatial (qualitatively interpreted) distributions in the whole proximal tibia of each taxon are summarized below. Here we assume that regions of high rBV/TV (red color) reflect the positions in which the proximal tibia is loaded most frequently and/or with high magnitude.

**FIGURE 3:**
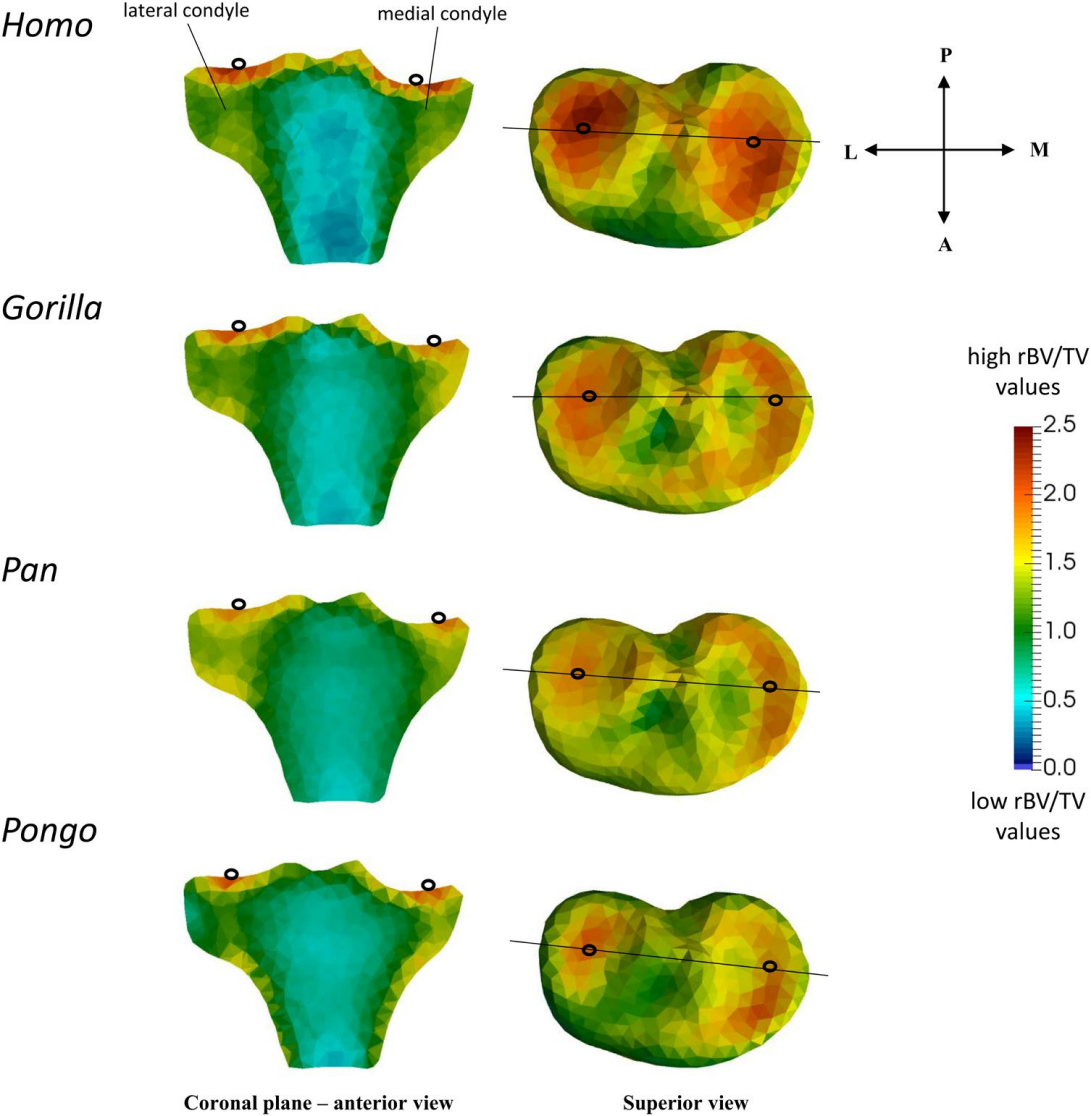
Coronal cross sections of the rBV/TV mean models of the tibial plateau and tibial condyles. Horizontal black lines through the superior view mean models show where the cross‐sectional coronal planes are positioned. Hollow black circles indicate section plane position. Red color shows the highest rBV/TV magnitude and blue color shows the lowest rBV/TV magnitude. L, lateral; M, medial; A, anterior; P, posterior. Posterior and anterior directions refer to the models in the superior view only.

In *Homo*, the highest rBV/TV values were found at the tibial plateau (Figure 3) within the center of both the medial and lateral condyles. The anterior region of the tibial plateau had low rBV/TV values. In non‐human apes, the highest rBV/TV values were found on the tibial plateau in the center of the lateral condyle and medially on the medial condyle (Figure 3). In *Pongo*, rBV/TV values were lowest at the latero‐anterior region of the tibial plateau compared with other taxa (Figure 3). In *Gorilla, Pan*, and *Pongo*, rBV/TV values were generally higher in the medial condyle compared to the lateral condyle, based on observed spatial distribution patterns. *Pongo* showed the greatest discrepancy in rBV/TV values between the medial and lateral condyles. In *Gorilla*, and to a lesser degree in *Pan*, high rBV/TV values extended deeply under both tibial condyles, while *Pongo* showed lower rBV/TV values in these regions (Figure 3).

**FIGURE 4:**
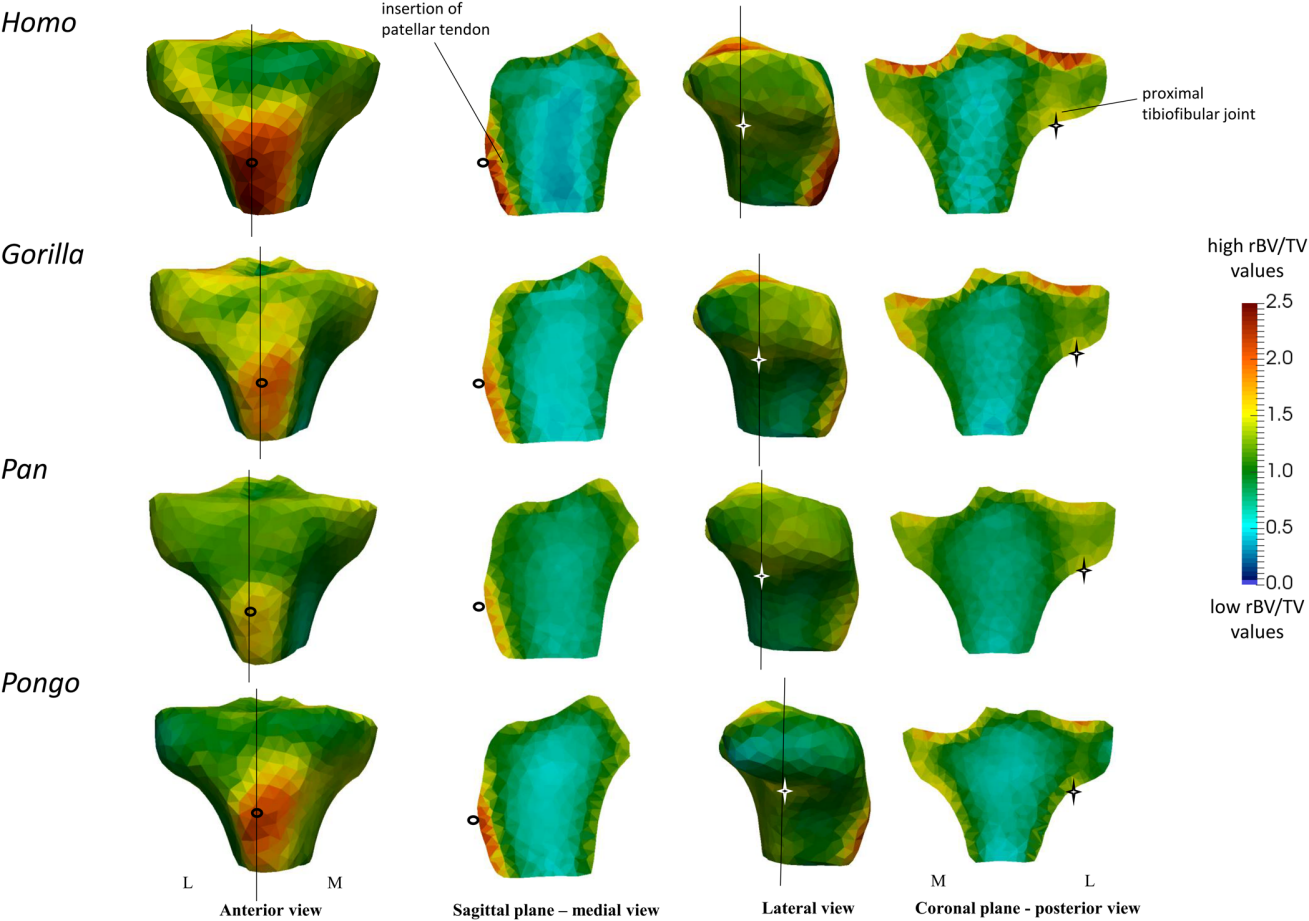
Sagittal (left‐middle) and coronal (right) cross sections of the rBV/TV mean models under the insertion of patellar ligament and under the tibiofibular joint. Vertical black lines in the anterior and lateral views show where the cross‐sectional coronal and sagittal planes are positioned. Hollow black circles and a black star indicate section plane position. Red color shows the highest rBV/TV magnitude and blue color shows the lowest rBV/TV magnitude. L, lateral; M, medial.

All studied taxa showed high rBV/TV under the insertion of the patellar ligament; however, this concentration did not extend deeply below the insertion site in any taxon (Figure 4). *Gorilla* showed the least variation under the presumed insertion of the patellar ligament across all taxa (see standard deviation values in Figure S1). Moreover, all taxa showed high rBV/TV values under the proximal tibiofibular joint (Figure 4). The rBV/TV values were highest in *Homo* and extended superiorly into the tibial plateau. However, in *Pan*, and especially in *Pongo*, high rBV/TV did not extend into the lateral condyle posteriorly as deeply, while *Gorilla* was intermediate between *Pan*/*Pongo* and *Homo* (Figure 4).

**FIGURE 5:**
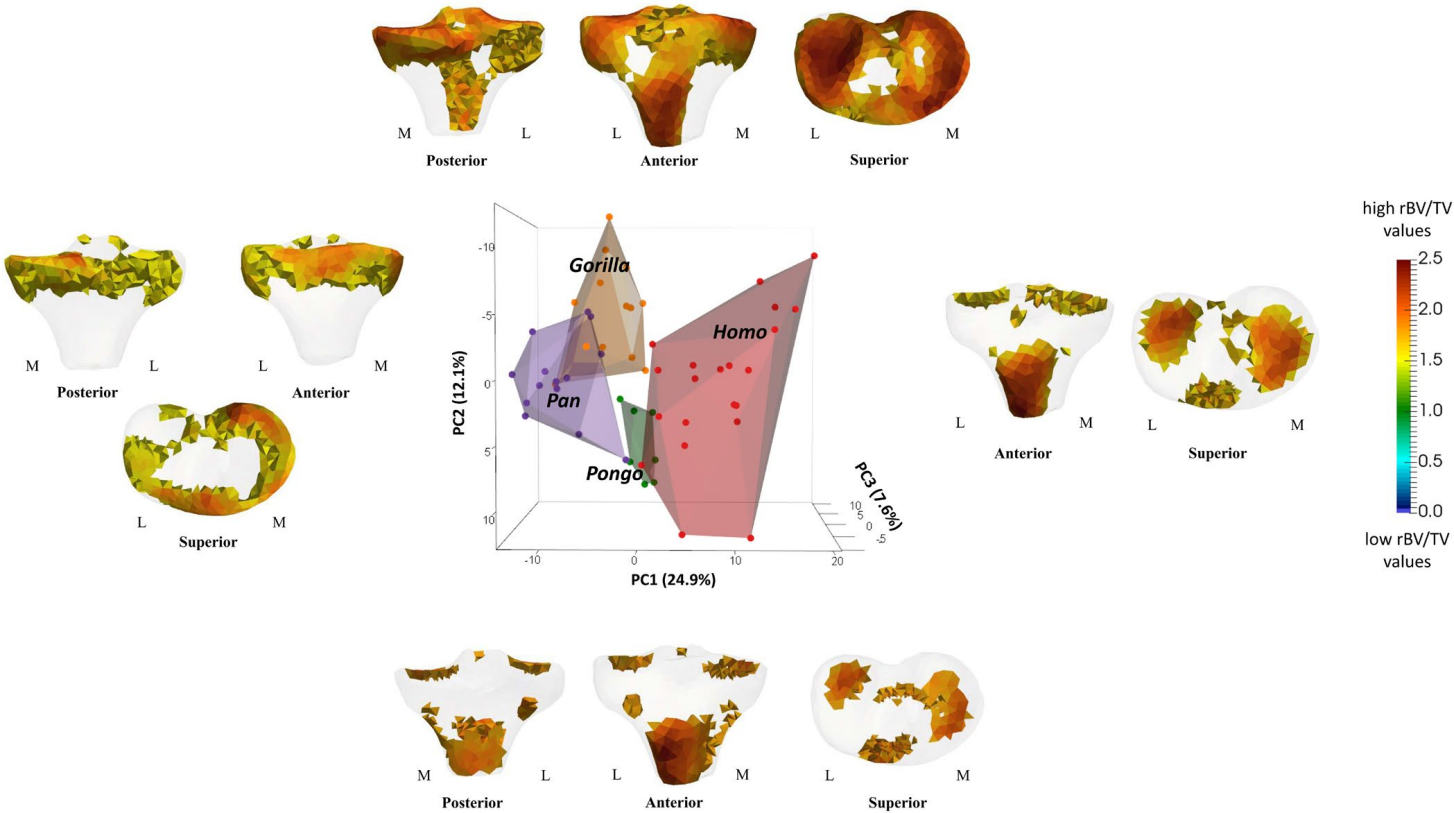
PCA of rBV/TV distribution in the proximal tibia. Thresholded models at each end of PC1 and PC2 identify the regions of high rBV/TV that are plus/minus 3 SD of variance along each axis. Humans are separated from non‐human apes along PC1 and *Pongo* is separated from *Gorilla* along PC2. L, lateral; M, medial.

PC1 explained 24.9% of the variation in rBV/TV and separated *Homo* from non‐human apes, with positive PC1 scores (see the loading models along PC1 axes in Figure 5) associated with higher rBV/TV in the middle of the lateral and medial condyles and under the insertion of patellar ligament [although rBV/TV was highly variable under the insertion of patellar ligament (Figure S1) in *Homo*]. Negative PC1 scores were associated with higher rBV/TV values found anteriorly on the tibial plateau and on the medial aspect of the medial condyle in non‐human apes (see the loading models along PC1 axes in Figure 5).

PC2 accounted for 12.1% of the variation in rBV/TV values and separated *Pongo* from *Gorilla* (Figure 5), due to a different spatial distribution beneath the tibial plateau and under the insertion of the patellar ligament. In *Pongo*, higher rBV/TV values were found in the posterior lateral condyle and along the anterior margin of the medial condyle, whereas in *Gorilla*, the highest rBV/TV values were along the posterior medial margin (see also Figure S2). In *Gorilla*, rBV/TV under the insertion of the patellar ligament extended posteriorly (reaching both sides of the tibial plateau), which differed from a distally located spatial distribution under this ligament in *Pongo*. PC3 accounted for 7.6% of the variation in rBV/TV values and separated *Pongo* from *Pan* due to a different spatial distribution on the tibial plateau and in the lateral condyle (see the loading models along PC3 axes in Figure S2). *Pan* showed homogeneous rBV/TV values across the lateral condyle and lacked a marked high rBV/TV anteriorly on the medial condyle. In contrast, *Pongo* rBV/TV values were found centrally on the lateral condyle and along the medial aspect of the medial condyle on the tibial plateau. *Pan* also showed higher rBV/TV values at the lateral condyle (negative PC3) compared to *Pongo* (see the loading models along PC3 axes in Figure S2). Permutational pairwise MANOVAs revealed no significant differences in rBV/TV PC scores, based on the first 3 PCs, between *Homo* and *Pongo* or between *Gorilla* and *Pan* (Table 2). All other pairwise comparisons were statistically significant. These results reflect group separation along the first three principal components, which represent the major axes of variation in the dataset (Figure 5; Figure S2).

#### 4.1.2 Quantitative Comparisons in the Cropped Tibial Plateau

**FIGURE 6:**
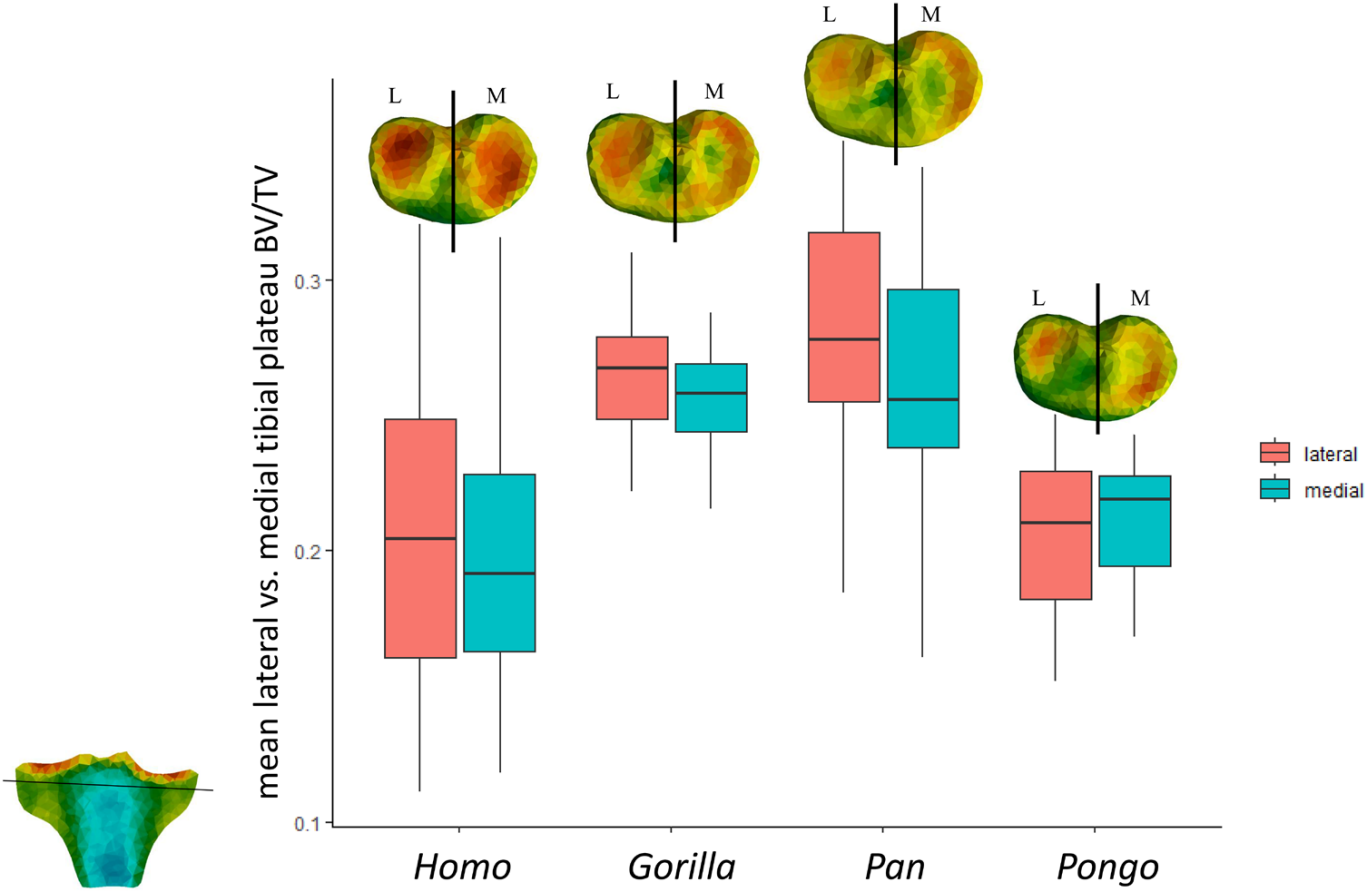
Mean tibial plateau BV/TV (measured as everything superior to the horizontal black line) by taxon. Black line shows where the medial and lateral condyle were divided. Mean BV/TV is greater in lateral side of tibial plateau compared to medial side in humans and greater in medial side of tibial plateau compared to lateral side in all non‐human apes. L, lateral; M, medial.

To complement the spatial distribution patterns of rBV/TV in the whole proximal tibia, we also compared mean absolute BV/TV values within a standardized cropped region of the tibial plateau (Figure 6). Dunn's tests showed mean absolute BV/TV in the tibial plateau to be significantly higher in *Gorilla* and *Pan* compared to *Homo* (*p*‐values < 0.001), in *Gorilla* compared to *Pongo* (*p*‐value = 0.008) and in *Pan* compared to *Pongo* (*p*‐value < 0.010; Table 3; Figure 6). *Gorilla* and *Pan* did not differ significantly from each other (*p*‐value = 0.999). *Homo* was found to have the lowest mean BV/TV and the most variable BV/TV statistical distribution in the tibial plateau and did not differ significantly from *Pongo* [*p*‐value = 0.999 (Table 3; Figure 6)]. In *Homo*, mean BV/TV in the lateral versus medial side of the tibial plateau was nonsignificantly different (Table 3), while the opposite pattern was found in non‐human apes where the mean BV/TV in the medial side of the tibial plateau was significantly higher than the lateral side (Figure 6; Table 3). In the lateral side only, mean BV/TV was significantly higher in *Gorilla* and *Pan* compared to *Homo* (*p*‐values < 0.003) and in *Gorilla* and *Pan* compared to *Pongo* (*p*‐values < 0.007). In the medial side only, mean BV/TV was significantly higher in *Gorilla* and *Pan* compared to *Homo* (*p*‐values < 0.001), in *Gorilla* compared to *Pongo* (*p*‐value = 0.010), and in *Pan* compared to *Pongo* [*p*‐value < 0.021 (Figure 6; Table 3)].

### 4.2 Degree of Anisotropy in the Whole Proximal Tibia

The statistical (quantitative) and spatial (qualitatively interpreted) distributions of DA in the whole proximal tibia of each taxon are summarized below. Here we assume that regions of high DA (red color) are associated with the joint positions in which the proximal tibia is the most stereotypically loaded.

**FIGURE 7:**
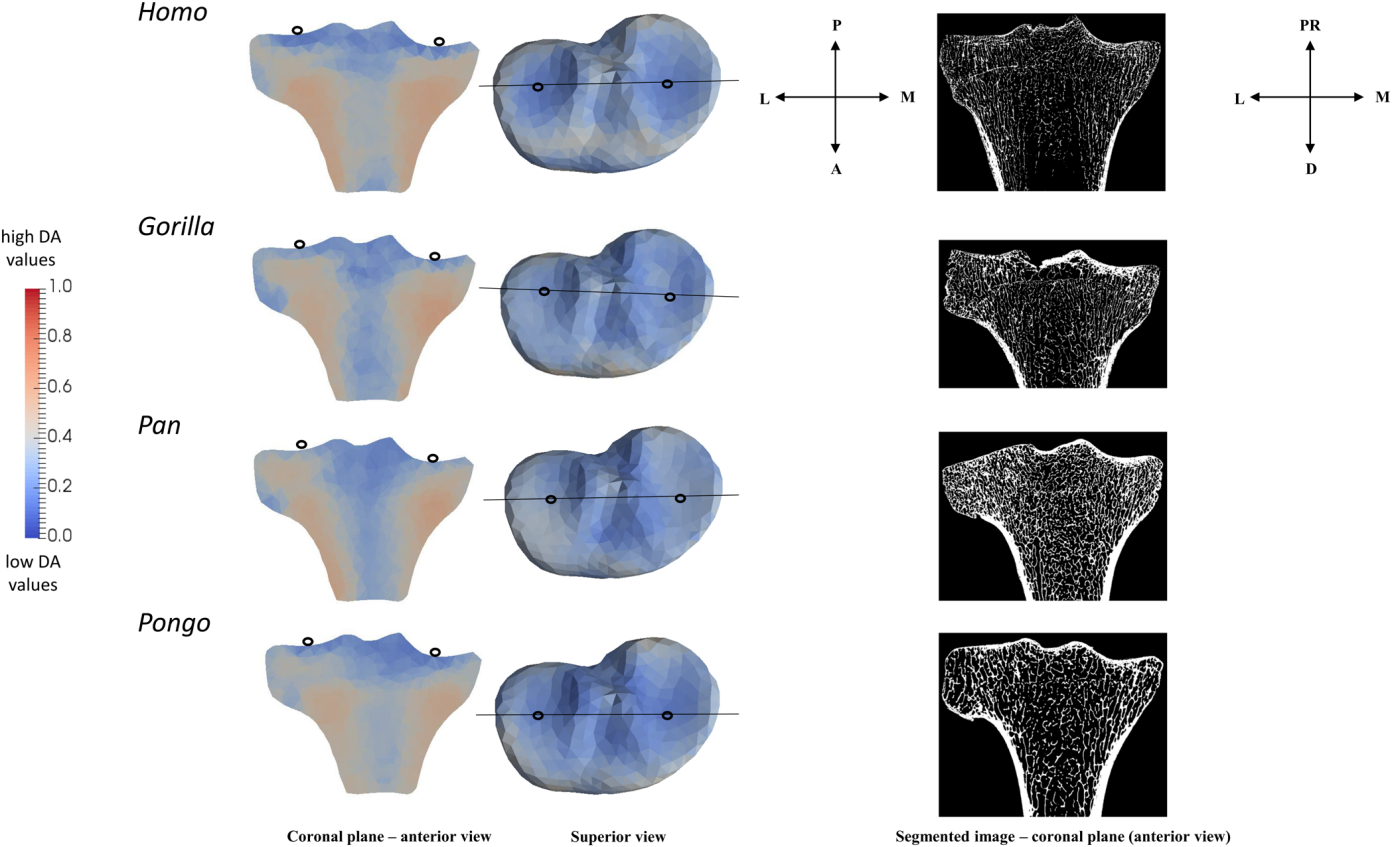
Coronal cross sections (left) of the DA mean models of the proximal tibia. Black lines in the superior view (middle) show where the cross‐sectional coronal planes and segmented microCT images (right) are positioned. Hollow black circles in the anterior and superior views indicate section plane position. Red color shows the highest DA magnitude and blue color shows the lowest DA magnitude. Orientation axes represent the orientation for the superior view and of the segmented images. L, lateral; M, medial; A, anterior; P, posterior; PR, proximal; D, distal.

**FIGURE 8:**
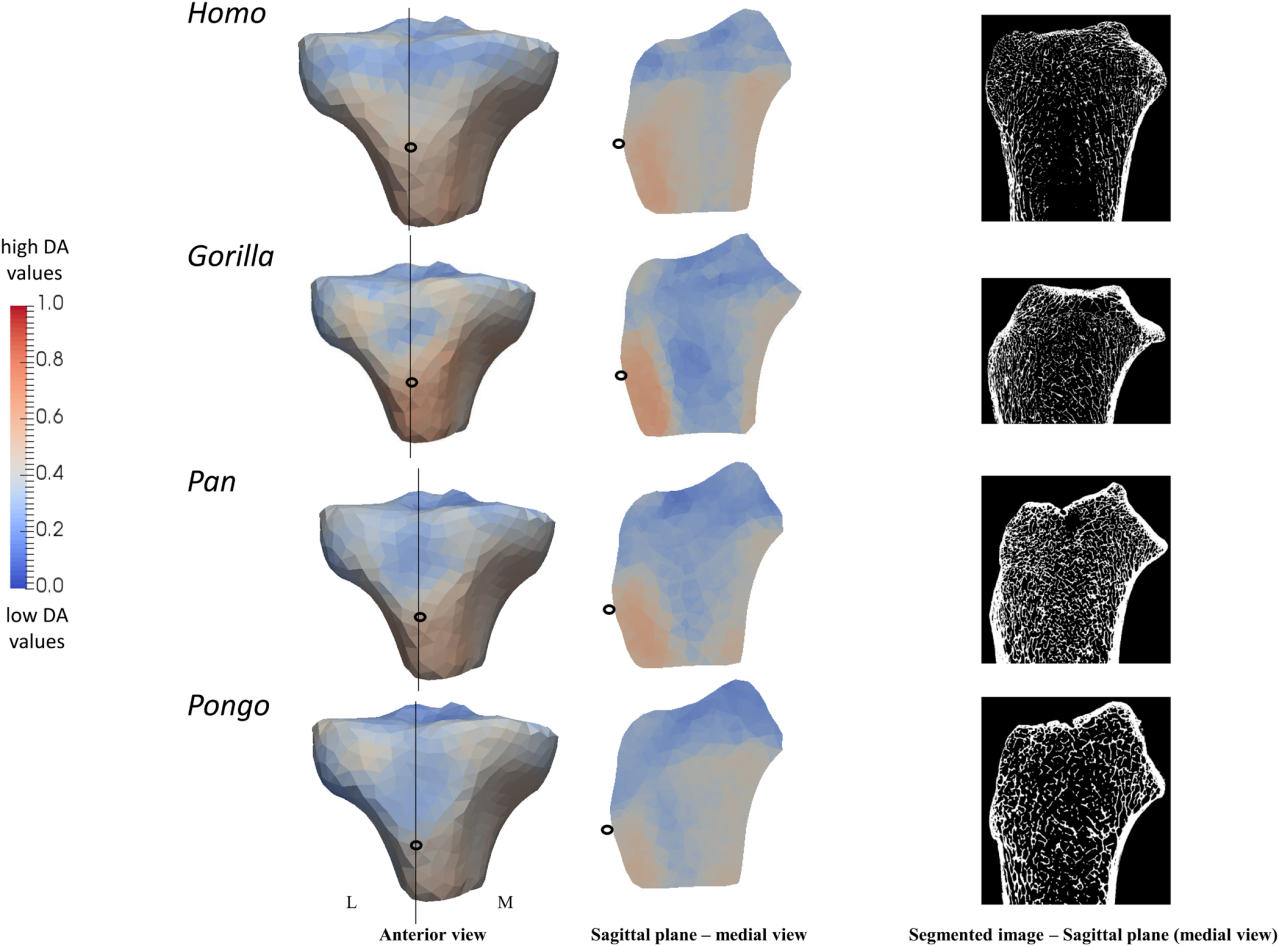
Sagittal cross sections (middle) of the DA mean models under the insertion of patellar ligament. Black lines in the anterior view (left) show where the cross‐sectional sagittal planes and segmented images are positioned. Hollow black circles in the anterior and medial views indicate section plane position. Red color shows the highest DA magnitude and blue color shows the lowest DA magnitude. L, lateral; M, medial.

We found a common pattern across all taxa of more isotropic values just beneath the tibial plateau but more anisotropic values deeper within the epiphysis (Figure 7). In *Homo*, DA values were more similar between the medial and lateral condyles and generally higher compared to non‐human apes (Figure 7). In contrast, in non‐human apes, we found higher DA values in the medial condyle compared to the lateral condyle (Figure 7). In *Homo* and *Pongo*, the anterior side of the medial condyle was most variable in DA values (Figure S3). We found high DA under the insertion of the patellar ligament in all taxa, with the highest values in *Gorilla* and the lowest in *Pongo* (Figure 8).

**FIGURE 9:**
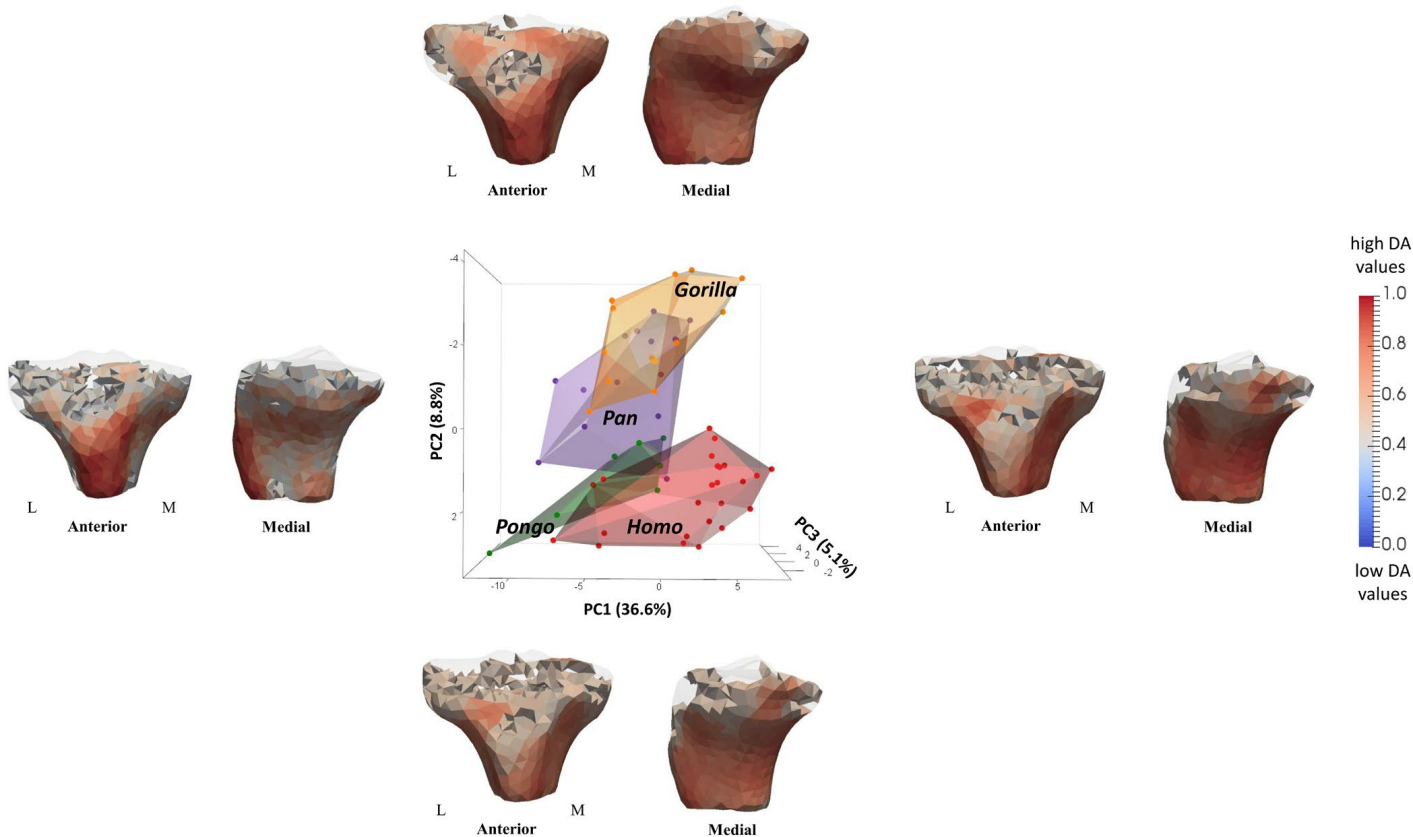
PCA of variation in DA among the study taxa. There is no separation between taxa on PC1, while *Gorilla* is separated from *Homo/Pongo* on PC2. Thresholded models at the end of each PC axis represent the regions of high DA variance along PC1 and PC2. L, lateral; M, medial.

**FIGURE 10:**
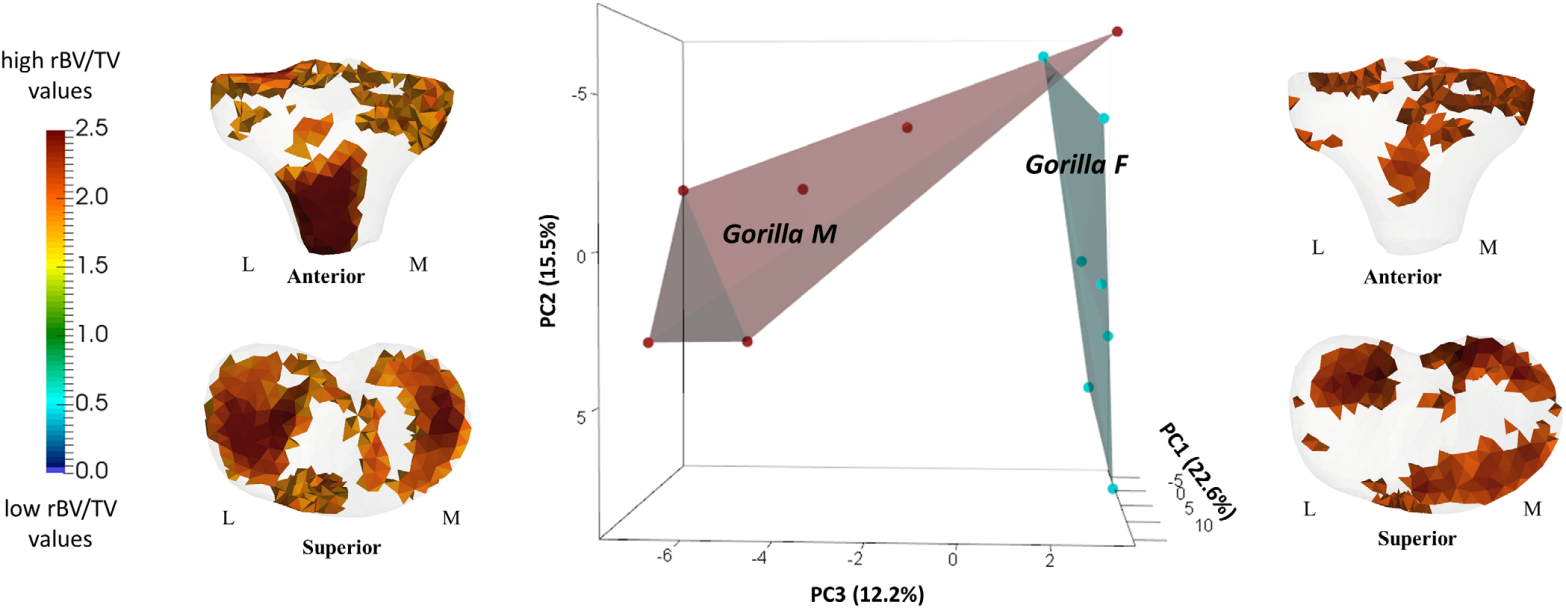
PCA of rBV/TV in the proximal tibia of *Gorilla* males and females. Thresholded models at each end of PC3 represent the regions of high rBV/TV that are plus/minus 3 SD along PC3, which broadly separates male and female *Gorilla* L, lateral; M, medial; F, female; M, male.

PC1 explained 36.6% of the variation in DA values at each mesh element and did not separate taxa from each other (Figure 9). PC2 accounted for 8.8% of the variation in DA values and separated *Gorilla* from *Homo* and *Pongo*. Negative PC2 distinguished *Gorilla* due to higher DA values under the insertion of patellar ligament (see the loading models along PC2 axes in Figure 9). *Pan* occupied an intermediate position along PC2, falling between the more distinct clusters of *Gorilla*, *Pongo*, and *Homo*. PC3 accounted for 5.1% of the variation in DA values and did not show any separation between studied taxa (Figure S4). Permutational pairwise MANOVAs revealed no significant differences in DA PC scores, based on the first 2 PCs, between *Homo* and *Pongo*, *Gorilla* and *Pan*, or *Pan* and *Pongo* (Table 2). All other pairwise comparisons were statistically significant. These results align with species separation patterns observed along the first two principal components, which summarize the major variation in the dataset (Figure 10).

### 4.3 Sex Differences in Mean Trabecular Architecture in the Whole Proximal Tibia

Female and male *Gorilla* did not separate completely on PC1 (explaining 22.6% of the variation) or on PC2 (explaining 15.5% of the variation) (Figure S5). However, most female and male specimens were distinguished from one another, with the variation that separates them distributed across the first three PCs, with PC3 accounting for 12.2% of the variation. Thresholded mean loading models showed that female *Gorilla* separated from males (positive PC3) due to higher rBV/TV values on the anterior side of the tibial plateau and due to the different spatial distribution on the posterior side of the medial aspect of the tibial plateau. In contrast, in male *Gorilla* (negative PC3), high rBV/TV values were more in the center of lateral tibial condyle and under the presumed insertion of patellar ligament (see the loading models along PC3 axes in Figure 10). On PC3, one male individual shows higher concentration on the anterior side of the tibial plateau compared to other male individuals in the sample. Thus, this male outlier plots on the positive PC3, overlapping with females. Additionally, high rBV/TV values penetrated deeper into the trabecular network of the lateral condyle in males compared to females (Figure S5). Permutational pairwise MANOVAs, based on the first four PCs, found no significant differences in rBV/TV PC scores between female and male *Gorilla* (Table 2). We found no separation in rBV/TV spatial distribution between sexes in *Pan* and *Homo* (Figure S6).

**FIGURE 11:**
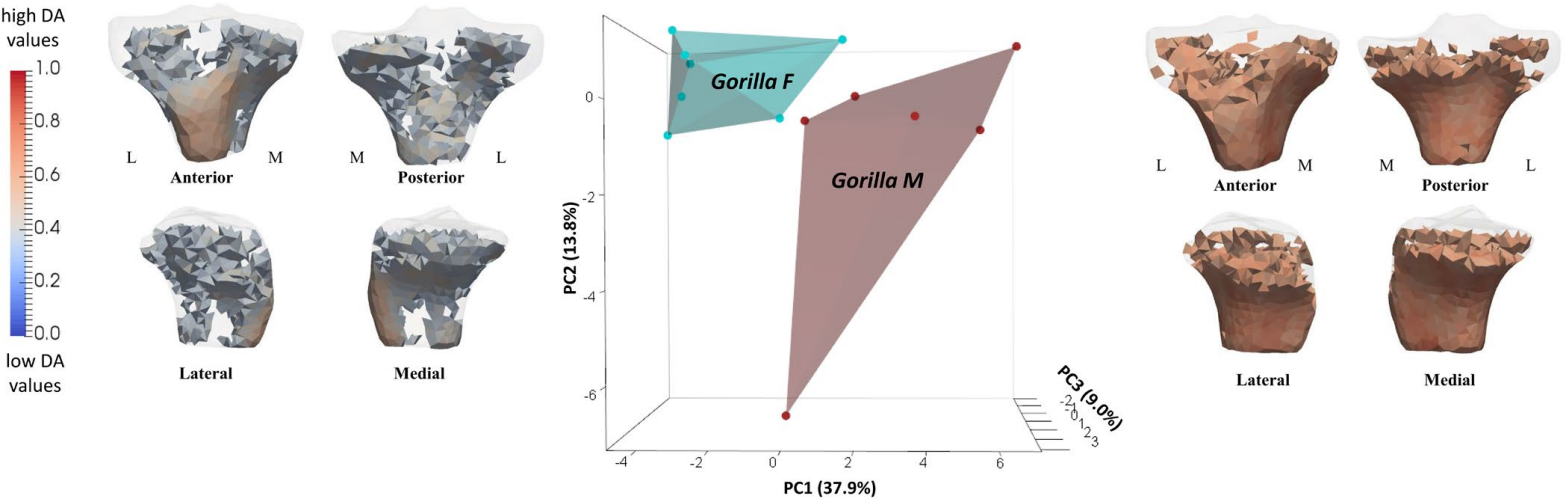
PCA of DA showing sex differences within *Gorilla* on PC1. Thresholded models at the end of each axis represent the regions of high DA that are plus/minus 3 SD along PC1. L, lateral; M, medial; F, female; M, male.

PC1 accounted for 37.9% of the variation in DA values. Negative PC1 distinguished female *Gorilla* due to lower DA values under the presumed insertion of the patellar ligament and in both condyles, and positive PC1 distinguished male *Gorilla* due to higher DA values inside both tibial condyles (see the loading models along PC1 axes in Figure 11). PC2 (explaining 13.8% of the variation) and PC3 (explaining 9% of the variation) did not show any separation with the separation of specimens on PC1 between female and male *Gorilla* (Figure S7). Permutational pairwise MANOVAs, based on the first two PCs, found significant differences in DA PC scores between female and male *Gorilla* (Table 2). We found no separation in DA spatial distribution between sexes in *Pan* and *Homo* (Figure S8).

## 5 Discussion

### 5.1 Trabecular Architecture Variation Among Humans and Non‐Human Apes

Our analysis of rBV/TV and BV/TV distribution in the proximal tibia supports key predictions of our hypotheses and reveals taxon‐specific differences in joint loading associated with locomotor behavior. Both, the spatial distribution of rBV/TV (from the whole proximal tibia) and BV/TV (from the cropped tibial plateau) highlight the loading regimes acting on the tibial plateau during habitual locomotion across taxa.

Our primary hypotheses predicted that *Homo* would differ from non‐human apes in having a trabecular structure of the proximal tibia that reflected habitual use of extended knee postures during bipedalism (see predictions 1A and 1E). These predictions were supported. We found high rBV/TV values in the anteroposterior center of each condyle on the tibial plateau and DA to be comparatively higher in *Homo* than in non‐human apes across both condyles. Some previous research has suggested that biomechanical loading through the medial and lateral femoral and tibial condyles are nearly equal in humans (e.g., Preuschoft 1971; Sylvester 2013), which is also supported by our results, while other studies have found differences between lateral and medial knee compartment loading (e.g., Holder et al. 2023; Kutzner et al. 2010; Mündermann et al. 2008). In *Homo*, mean BV/TV in the lateral tibial plateau was slightly (though not significantly) higher than in the medial side, whereas in non‐human apes, the medial side had significantly higher values. This suggests that in humans, the lateral condyle may bear greater stress during bipedal walking, potentially due to the valgus knee angle. However, considering that the medial condyle has a slightly larger articular surface, and that BV/TV differences are small, loading may still be relatively balanced between compartments.

Although humans are obligate bipeds, recent populations exhibit generally low BV/TV in both upper and lower limbs (which is supported by our results), likely reflecting reduced biomechanical loading from sedentism compared to more mobile Pleistocene and Holocene populations (Chirchir et al. 2015; DeMars et al. 2021; Ryan and Shaw 2015). This trend appears systemic across both forager and agricultural groups (Saers et al. 2016), and its causes may be multifactorial, possibly involving systemic reductions in upper limb loading, dietary shifts, disease prevalence, hormonal influences, or other biological factors (Chavassieux et al. 2007; Chirchir 2015, 2021; Dawson‐Hughes et al. 1997; Riggs and Melton 1986; Robling et al. 2006; Weber 2020; Tsegai et al. 2018).

In general, the trabecular architecture of the human proximal tibia was found to be the most consistent with biomechanical loading during bipedal locomotion and with a stereotypically extended knee position during all phases of human walking. The trabecular structure of the human proximal tibia is also consistent with that of the distal femur (Lukova et al. 2024a). In our previous study, we found high rBV/TV values in the posteroinferior regions of both femoral condyles, reflecting more extended knee postures during all gait phases of walking compared to non‐human apes. We also found higher rBV/TV values in the lateral femoral condyle compared to medial condyle. We suggested that this pattern in the distal femur was consistent with the extended knee posture combined with a valgus knee angle (Lukova et al. 2024a).

In contrast, the hypothesis that *Pan* and *Gorilla* would differ from humans and *Pongo* in having trabecular structure of the proximal tibia that reflected habitual use of flexed knee postures during terrestrial and arboreal locomotion (see predictions 1B and 1E) was supported. We found rBV/TV to be higher in the medial condyle and on the medial side of the tibial plateau in *Pan* and *Gorilla* compared to humans and *Pongo*. However, we did not find any significant differences between *Gorilla* and *Pan* as also predicted. In African apes, the highest rBV/TV values were concentrated along the medial edge of the medial condyle, while rBV/TV was highest in the center of lateral condyle. This rBV/TV pattern in *Pan* and *Gorilla* is consistent with higher medial knee compartment loading and flexed knee postures during locomotion, distinguishing the African apes from *Homo* and *Pongo*. This suggests that during flexed knee locomotion, the medial femoral condyle presses on the medial tibial margin, inducing trabecular (re)modeling along the medial edge. In both taxa, high DA values were found across both condyles and low DA values were found on the tibial plateau.

Moreover, the single attachment of the lateral meniscus in African apes permits greater internal/external rotation compared to the double attachment of the more stable medial meniscus (Tardieu 1981). Coupled with a varus knee angle, this results in a rotational axis passing through the medial condyle and a concentration of forces in that compartment (Churchill et al. 1998; Freeman and Pinskerova 2005; Schipplein and Andriacchi 1991; Sylvester 2013). The resulting high medial BV/TV values in African apes likely reflect this combination of rotational and compressive loading during flexed postures. Tardieu (1981) also reported greater knee rotation range in *Pan* (and likely *Gorilla*) than in humans, likely advantageous during frequent arboreal locomotion (Doran 1997; Remis 1995, 1998). Supporting this, human kinematic studies have shown that deep knee flexion is associated with internal/external rotation and increased tibiofemoral forces, up to 5.6 times body weight mediolaterally and 3.5 times anteroposteriorly (Dahlkvist et al. 1982). These forces exceed those during walking (Mikosz et al. 1988; Morrison 1970; Schipplein and Andriacchi 1991; Seireg and Arvikar 1975; Taylor et al. 2004; Wang et al. 2014), and also increase stress on the patellar ligament and posterior tibial plateau (Nagura et al. 2002). Assuming comparable stress patterns in apes during flexion, our results are consistent with expected trabecular (re)modeling, particularly elevated BV/TV beneath the patellar ligament and along the posterior and medial margins of the medial condyle due to varus knee alignment.

Changes in knee angle influence joint reaction forces and contact area, with more flexed postures leading to higher articular stress (Kutzner et al. 2010; Taylor et al. 2004) and increased posterior condylar contact (Von Eisenhart‐Rothe et al. 2004). In general, the trabecular structure of the proximal tibia in African apes is also consistent with high rBV/TV values posterosuperiorly in both femoral condyles and mediolaterally on the patellar articulation of the femur, which also reflects loading of the distal femur in a flexed knee posture (Lukova et al. 2024a).

Our initial hypotheses predicted that *Pongo* would significantly differ from other non‐human apes due to more variable knee postures and knee loading during arboreal locomotion, as well as their higher medial knee compartment loading associated with a varus knee angle (predictions 1C and 1E). These predictions were partially supported. We found rBV/TV to be concentrated medially on the medial side of the tibial plateau, as in African apes; however, this concentration extended more anteriorly in *Pongo*. We suggest that this pattern is consistent with loading of the knee in extended as well as flexed knee postures while the medial knee compartment is loaded more than the lateral knee compartment. Specifically, this pattern could suggest that when the knee is flexed and more medially loaded, high rBV/TV values are concentrated proximally on the medial side of the tibial plateau. Similarly, the more the knee is extended we would predict more loading resulting in denser anterior trabecular structure. Thus, this pattern may signal higher knee extension in *Pongo* compared to African apes. While overall the trabecular architecture of the proximal tibia in *Pongo* suggests loading during flexed postures, the differences in spatial distribution across the tibial plateau imply a distinct loading regime from *Pan* and *Gorilla*. A previous study of trabecular architecture in the distal femur found that *Pongo* did not differ significantly from *Pan*, but did separate from *Gorilla* (Georgiou et al. 2019). However, our more recent study of trabecular architecture in distal femora, which incorporated the more quantitative and statistical tools of cHMA, found a greater degree of separation between both *Pongo* and *Pan*, and *Pongo* and *Gorilla* (Lukova et al. 2024a).

Like African apes, *Pongo* possesses a lateral meniscus with a single attachment site, which permits a greater range of knee rotation and posture variability (Girgis et al. 1975). This anatomical configuration may contribute to the more homogenous BV/TV distribution between the medial and lateral sides of the tibial plateau seen in *Pongo*, contrasting with the more asymmetrical loading patterns in African apes. Our results support this, showing significantly higher BV/TV on the medial side of the tibial plateau in *Pongo*, similar to *Pan* and *Gorilla*, yet differing from them in the spatial pattern of trabecular distribution. Interestingly, the pattern of loading in *Pongo* more closely resembles that of *Homo* than that of African apes, with both *Pongo* and *Homo* exhibiting lower overall BV/TV values in the tibial plateau and a more evenly distributed loading pattern. This may reflect a shared characteristic of more variable or lower magnitude loading associated with increased postural range and behavioral flexibility. However, due to the limited sample size for *Pongo*, we could not statistically confirm significant differences from *Homo*.

Our final prediction (1D) for the first hypothesis was also supported. In *Homo*, the patellar ligament plays a crucial role in the biomechanics of the knee joint (DeFrate et al. 2007; Halonen et al. 2016; Taylor et al. 2004). The patellar ligament helps to stabilize the knee joint by fixing the patella in place. When the quadriceps contract, it pulls on the patella via the quadriceps tendon, which in turn pulls on the patellar ligament (e.g., Grelsamer and Klein 1998; Hehne 1990; Ramsey and Wretenberg 1999). This transmission of force from the quadriceps muscles to the tibia is substantial during activities such as walking, running, jumping, and squatting (Nilsson and Thorstensson 1989; Racic et al. 2009; Stäubli et al. 1996). In both humans and non‐human apes, contraction of the quadriceps exerts force through the quadriceps tendon, pulling on the patella and transmitting force through the patellar ligament, which helps to align the patella with the long axis of the lower limb, an arrangement crucial for stabilizing the knee joint and ensuring effective function during movement (e.g., Hart et al. 2023; Lovejoy 2007; Payne et al. 2006; Preuschoft and Tardieu 1996). This force is transmitted through the patellar ligament to the tibia, enabling knee extension. Non‐human apes experience dynamic loading of the patellar ligament during various activities but especially during the activities where the knee is in deep flexion (D'Août et al. 2002; Sylvester 2013). The mechanical importance of the patellar ligament is reflected in our results as we found high rBV/TV values under the patellar ligament attachment in all taxa.

In *Homo*, the proximal fibula needs to resist repeated forces transmitted through the tibiofibular joints during bipedalism (Lambert 1971; Ogden 1974; Pietrobelli et al. 2023; Preuschoft 1971) and thus the articulation is less mobile compared to non‐human apes (Eichenblat and Nathan 1983; Ogden 1974; Pietrobelli et al. 2023; Sarma et al. 2015). This more stable articulation is reflected in our results, as the rBV/TV under the presumed proximal tibiofibular joint is the highest in *Homo* compared to the remaining taxa of our sample. The proximal tibiofibular articulation in non‐human apes is more mobile compared to humans, which may contribute to overall knee limb flexibility during arboreal locomotion (DeSilva 2009). While ankle dorsiflexion itself occurs at the talocrural joint and is not directly controlled by the proximal tibiofibular joint, since dorsiflexor muscles do not cross it, the increased mobility of the proximal tibiofibular joint may help accommodate the mechanical demands and force transmission associated with dorsiflexion during activities like vertical climbing. Earlier studies suggested that *Pan* uses relatively high ankle joint dorsiflexion compared to humans across various modes of locomotion (DeSilva 2009), and more recent research by Holowka et al. (2017) demonstrated that *Pan* exhibits dorsiflexion capabilities that are more similar to humans, including during vertical climbing. This closer similarity has been further supported by Venkataraman et al. (2013), who found comparable dorsiflexion abilities in human climbers facilitated by changes in soft tissues. Additionally, previous studies have shown that *Pan* has greater flexibility in the tibiofibular joint compared to *Gorilla* and *Pongo* (Crompton et al. 2010). This increased flexibility has been linked to the need to prevent anterior displacement of the fibula and accommodate wider bicondylar angles during knee flexion (Lovejoy 2007), as well as the need in *Pongo* to stabilize the knee during extension while walking bipedally in the trees (Thorpe et al. 2009). However, we were not able to find any significant differences between non‐human apes under the proximal tibiofibular joint that could reflect differences in mobility found in previous studies.

### 5.2 Sex Differences in Trabecular Architecture in *Homo*, *Gorilla*, and *Pan*

In our second hypothesis, we predicted that trabecular structure would reflect sex differences in locomotor behaviors of *Gorilla* but not that of *Pan* and *Homo*. Our prediction 2A that *Gorilla* would show sex differences due to a different level of arboreality was supported. Male *Gorilla*, rather than females, exhibited significantly higher rBV/TV values in the lateral condyle. Additionally, females showed higher rBV/TV values anteriorly on the medial side of the tibial plateau and lower rBV/TV under the tibial tuberosity compared with male *Gorilla*. We found DA values to be comparatively higher in both tibial condyles in male *Gorilla* compared with females.

These sex differences may be explained, at least in part, by anatomical differences. The medial tibial condyle of the male *Gorilla* is more concave compared to that of females (as in *Pan*) possibly due to the differences in body mass and/or differences in locomotor behavior (Sylvester 2013), which could potentially allow female *Gorilla* to have a greater range of knee motion. Partial separation between female and male *Gorilla* in our PCA results suggested a more extended (and flexed) knee postures, along with higher medial knee compartment loading (compared to lateral knee compartment loading) and a less stereotypically loaded proximal tibia in females compared to males. A higher level of knee extension and a generally higher range of motion at the knee was previously found in captive adult female *Gorilla* compared to males, particularly during vertical climbing (Isler 2005). This was supported by our recent examination of trabecular structure in the distal femur, with females showing higher rBV/TV values in the posterior regions of the lateral condyle, laterally on the patellar surface, and medially above the intercondylar fossa (Lukova et al. 2024a). In contrast, *Gorilla* males had higher rBV/TV values in the medial condyle (Lukova et al. 2024a). Compared to males, wild female *Gorilla* are more arboreal but also smaller in body mass (Remis 1997). *Homo* body size dimorphism approaches that of *Pan* (Smith and Jungers 1997); however, we did not find any sex differences in proximal tibia loading in these taxa (although small sample sizes necessitate additional examination of this finding). Thus, sex differences found in the proximal tibia in *Gorilla* might be driven by differences in body mass, the degree of arboreality, and/or by differences in knee loading. While our findings focus on the tibia, previous research has identified taxon‐based differences in trabecular architecture in other skeletal elements of *Gorilla*, such as the calcaneus (Harper and Patel 2024), suggesting that similar differences may also exist in the knee joint. However, further investigation of *Gorilla* locomotor kinematics and kinetics, particularly in wild communities, and larger skeletal samples are needed to address whether sex differences in trabecular structure exist in *Gorilla*.

## 6 Conclusion

This study is an examination of trabecular bone architecture within the hominid proximal tibia. Trabecular architecture in *Homo* indicates habitual use of extended knee postures during bipedalism and significantly differs from *Gorilla* and *Pan*. However, despite *Homo* loading only the lower limb during locomotion, mean BV/TV was significantly lower on the tibial plateau than that of non‐human apes. Trabecular architecture of *Gorilla* and *Pan* indicates higher medial knee compartment loading and use of flexed knee posture during terrestrial and arboreal locomotion and significantly differs from *Homo* and *Pongo*. Trabecular architecture of the proximal tibia suggests a greater degree of knee extension in *Pongo* compared to African apes and in male *Gorilla* compared to female *Gorilla*. Trabecular structure is not substantially different between sexes in *Pan* or *Homo*, reflecting greater presumed similarity in proximal tibia loading between sexes in these taxa. This study offers a comparative context of trabecular structure in the hominoid proximal tibia and can contribute to future studies of locomotion in extinct taxa.

## Author Contributions

**Andrea Lukova:** conceptualization, investigation, writing – original draft, methodology, validation, visualization, writing – review and editing, formal analysis. **Sebastian Bachmann:** methodology, formal analysis, software, writing – review and editing. **Alexander Synek:** methodology, formal analysis, software, writing – review and editing. **Dieter H. Pahr:** methodology, software. **Brandon Kilbourne:** writing – review and editing, resources. **Christopher J. Dunmore:** methodology, validation, writing – review and editing, formal analysis. **Tracy L. Kivell:** supervision, data curation, writing – review and editing, validation. **Matthew M. Skinner:** validation, writing – review and editing, data curation, supervision, funding acquisition.

## Supporting information


**Figure S1** Standard deviation maps of rBV/TV values in the proximal tibia of *Homo, Gorilla, Pan*, and *Pongo*. Vertical and horizontal lines through the SD models show where the cross‐sectional sagittal and coronal planes are positioned. Red color shows the highest variability in the rBV/TV values and blue color shows the lowest variability in the rBV/TV values. L, lateral; M, medial.

**Figure S2** PC3 of rBV/TV distribution in proximal femur of *Homo*, *Gorilla*, *Pan*, and *Pongo* showing separation among studied taxa. Models at the end of each axis represent the regions of high rBV/TV driving variance along PC3. Models demonstrate the rBV/TV values separating between *Pongo* (positive PC3 + 3SD) and *Pan* (negative PC3‐3SD). L, lateral; M, medial.

**Figure S3** Standard deviation maps of DA distribution of the proximal tibia of *Homo, Gorilla, Pan*, and *Pongo*. Red color shows the highest variability in the DA values and blue color shows the lowest variability in the DA values. L, lateral; M, medial.

**Figure S4** PCA of DA distribution in the proximal tibia of *Homo*, *Gorilla*, *Pan*, and *Pongo* on PC3.

**Figure S5** *Gorilla* mean models and PCA of rBV/TV distribution in the proximal tibia of *Gorilla* showing no separation on PC1 and partial separation on PC2 and PC3. Horizontal lines through the superior view mean models show where the cross‐sectional coronal planes are positioned. Circles in the superior and anterior views represent the homologous locations. F, female; M, male.

**Figure S6** PCA of rBV/TV distribution in the proximal tibia of (A) *Pan* and (B) *Homo* showing no separation between sexes. F, female; M, male.

**Figure S7** PCA of DA distribution in proximal tibia of *Gorilla* showing no separation between sexes on PC2 and PC3. F, female; M, male.

**Figure S8** PCA of DA distribution in proximal tibia of (A) *Pan*, and (B) *Homo* showing no separation between sexes. F, female; M, male.

**Table S1** Complete information of sample composition.
**Table S2:** Descriptive statistics of BV/TV distribution in tibial plateau.

## Data Availability

The data that support the findings of this study are openly available on the Human fossil record website at (human‐fossil‐record.org).
