# Supplementary figures and images for: Trabecular Architecture of the Proximal Tibia in Extant Hominids
Source: Am J Biol Anthropol. 2025 Jun 30;187(3):e70084. doi: 10.1002/ajpa.70084 (PMC12207363; doi:10.1002/ajpa.70084)

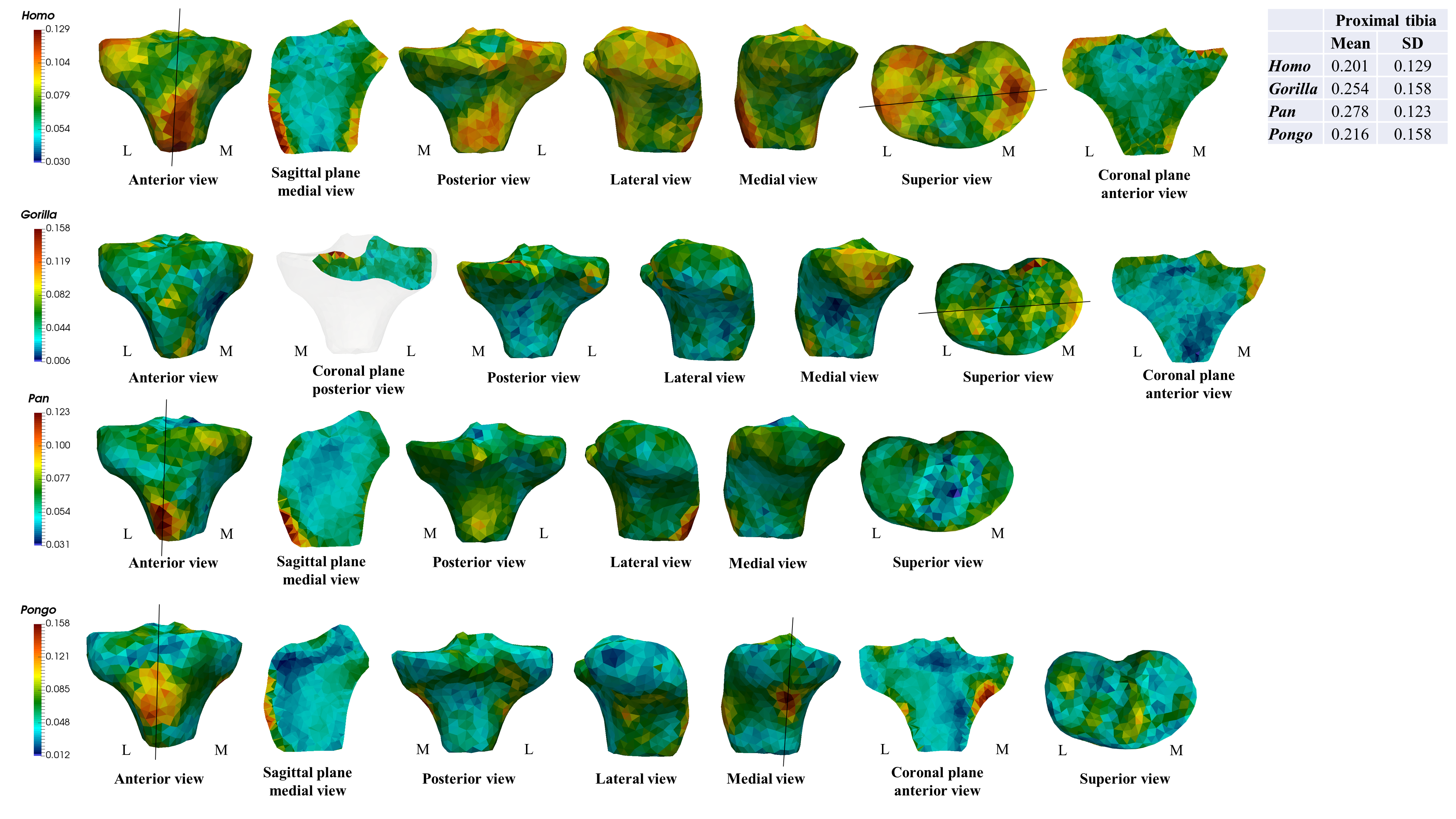

Supplement: Supplementary file 1 — Figure S1 Standard deviation maps of rBV/TV values in the proximal tibia of Homo, Gorilla, Pan, and Pongo. Vertical and horizontal lines through the SD models show where the cross‐sectional sagittal and coronal planes are positioned. Red color shows the highest variability in the rBV/TV values and blue color shows the lowest variability in the rBV/TV values. L, lateral; M, medial. [file AJPA-187-e70084-s005.tif]

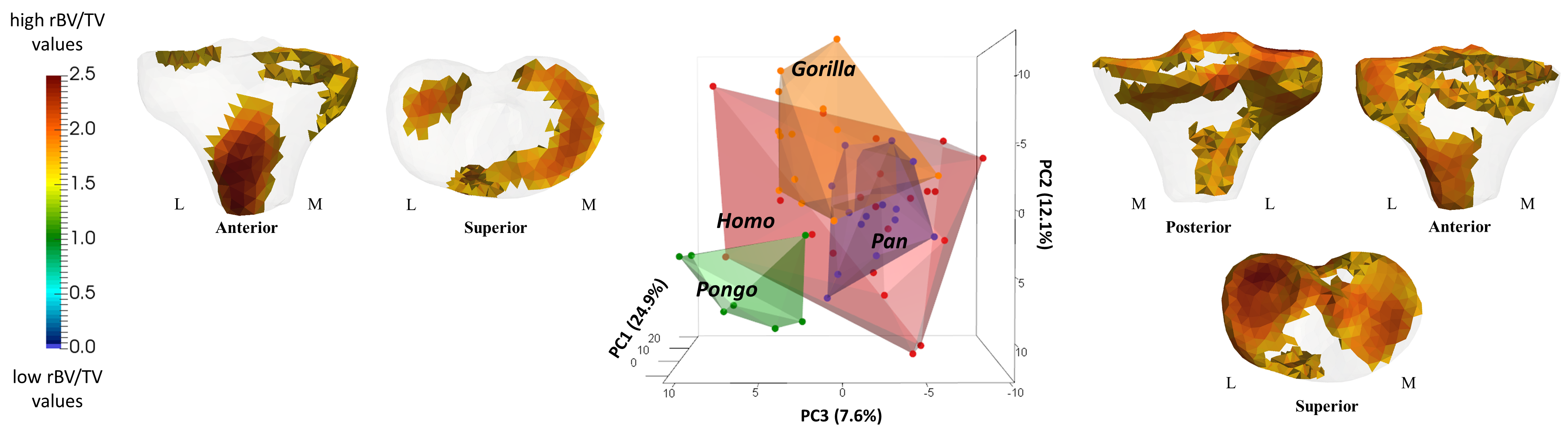

Supplement: Supplementary file 2 — Figure S2 PC3 of rBV/TV distribution in proximal femur of Homo, Gorilla, Pan, and Pongo showing separation among studied taxa. Models at the end of each axis represent the regions of high rBV/TV driving variance along PC3. Models demonstrate the rBV/TV values separating between Pongo (positive PC3 + 3SD) and Pan (negative PC3‐3SD). L, lateral; M, medial. [file AJPA-187-e70084-s006.tif]

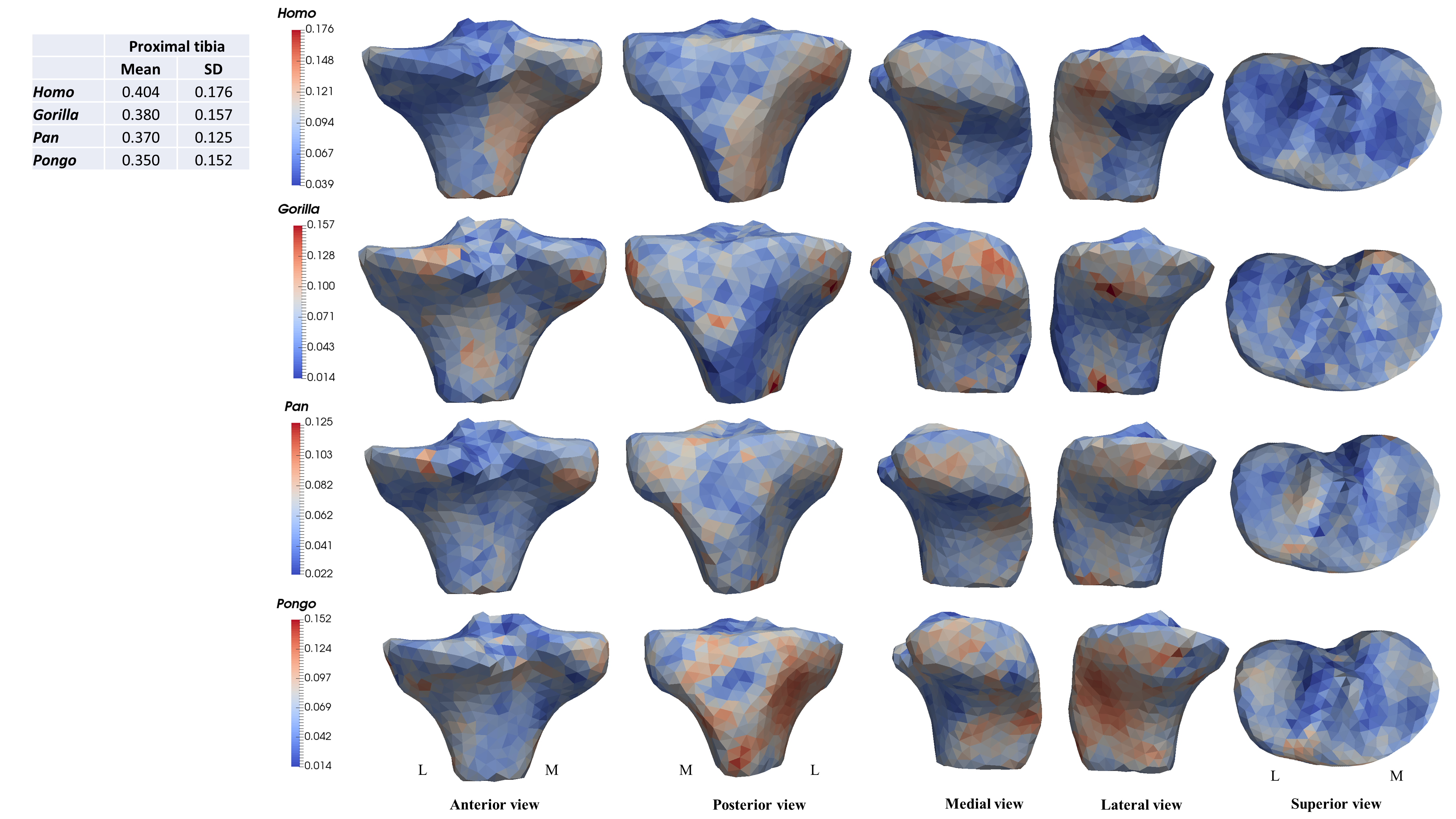

Supplement: Supplementary file 3 — Figure S3 Standard deviation maps of DA distribution of the proximal tibia of Homo, Gorilla, Pan, and Pongo. Red color shows the highest variability in the DA values and blue color shows the lowest variability in the DA values. L, lateral; M, medial. [file AJPA-187-e70084-s003.tif]

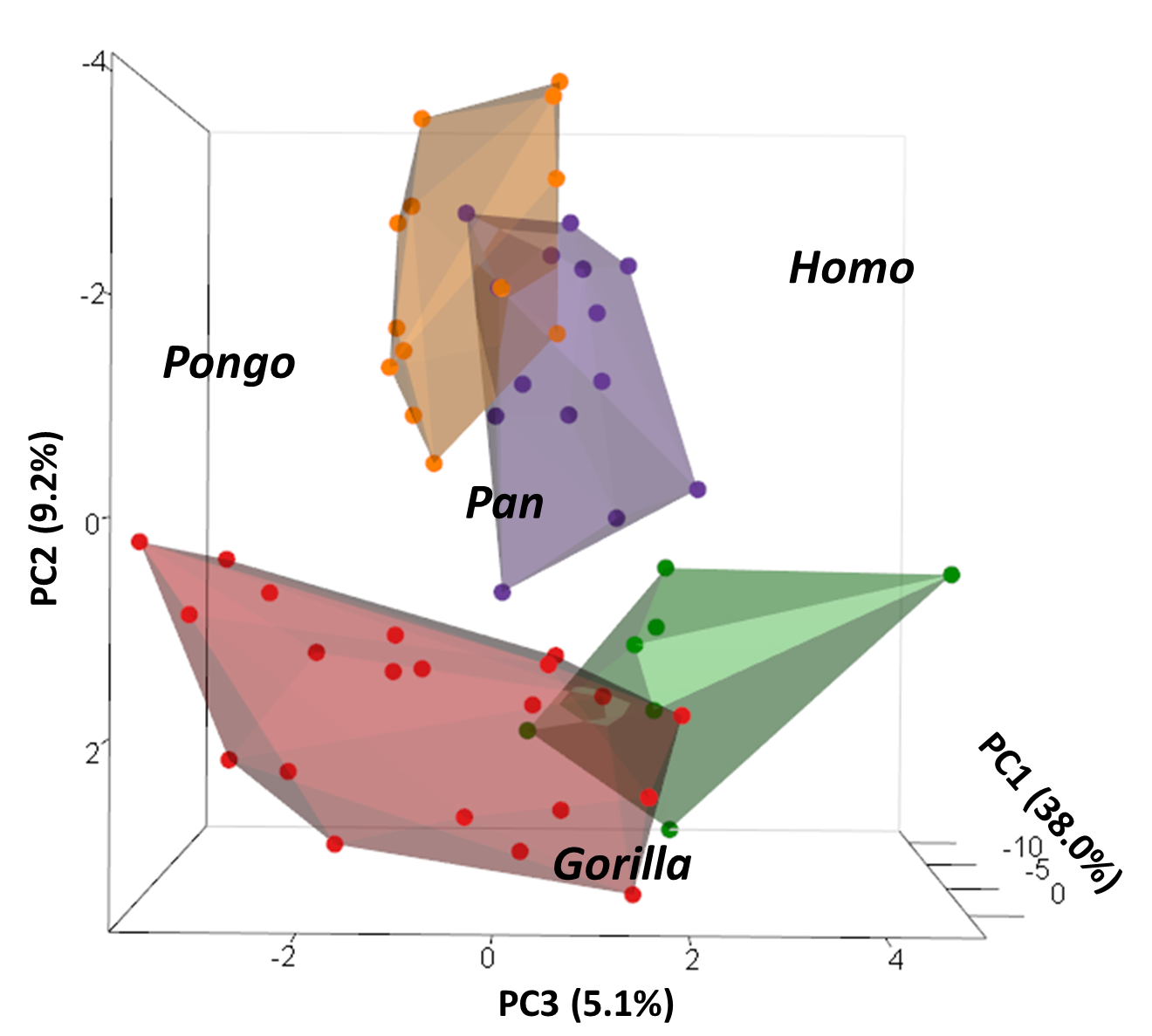

Supplement: Supplementary file 4 — Figure S4 PCA of DA distribution in the proximal tibia of Homo, Gorilla, Pan, and Pongo on PC3. [file AJPA-187-e70084-s004.tif]

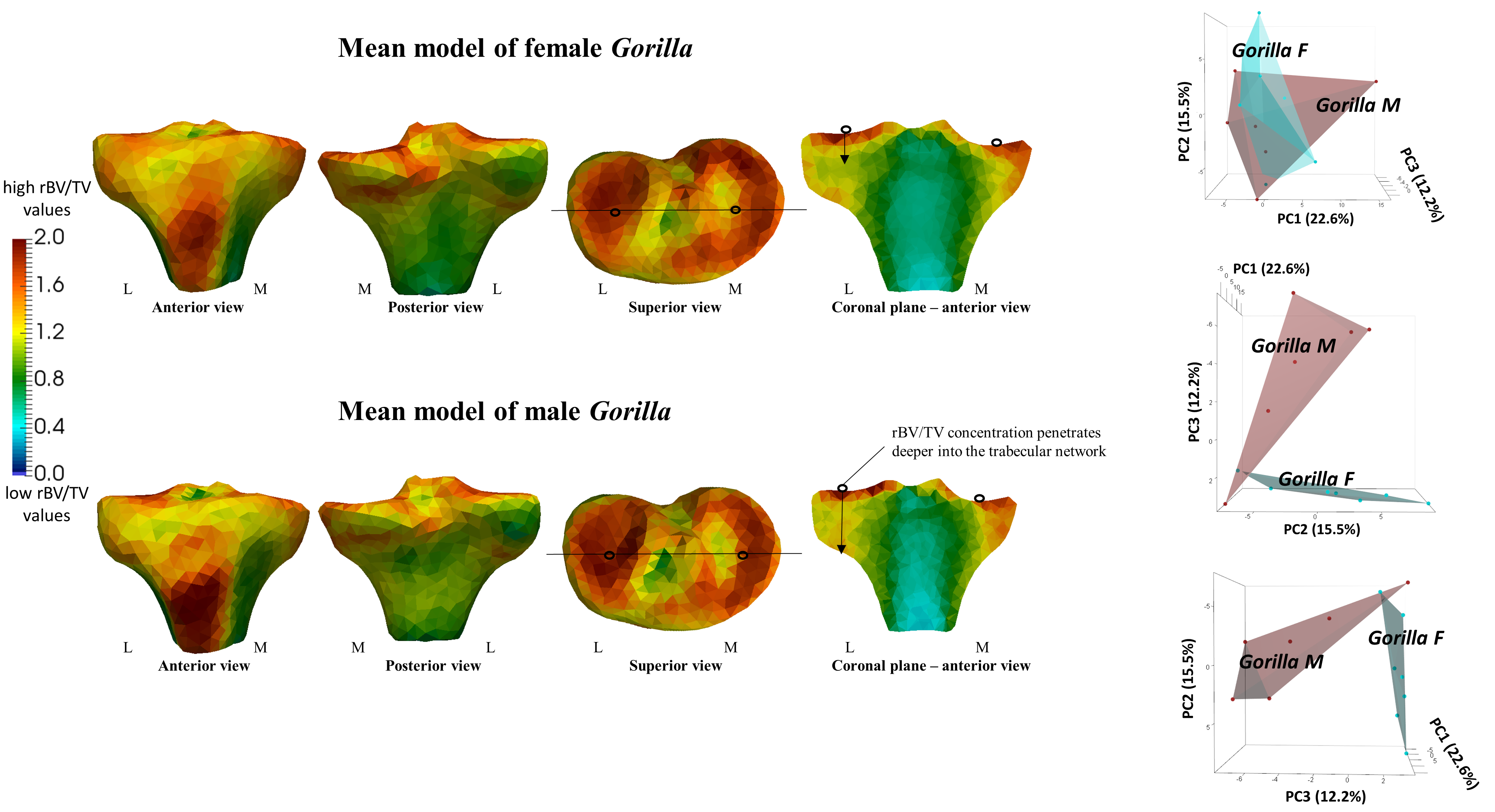

Supplement: Supplementary file 5 — Figure S5 Gorilla mean models and PCA of rBV/TV distribution in the proximal tibia of Gorilla showing no separation on PC1 and partial separation on PC2 and PC3. Horizontal lines through the superior view mean models show where the cross‐sectional coronal planes are positioned. Circles in the superior and anterior views represent the homologous locations. F, female; M, male. [file AJPA-187-e70084-s008.tif]

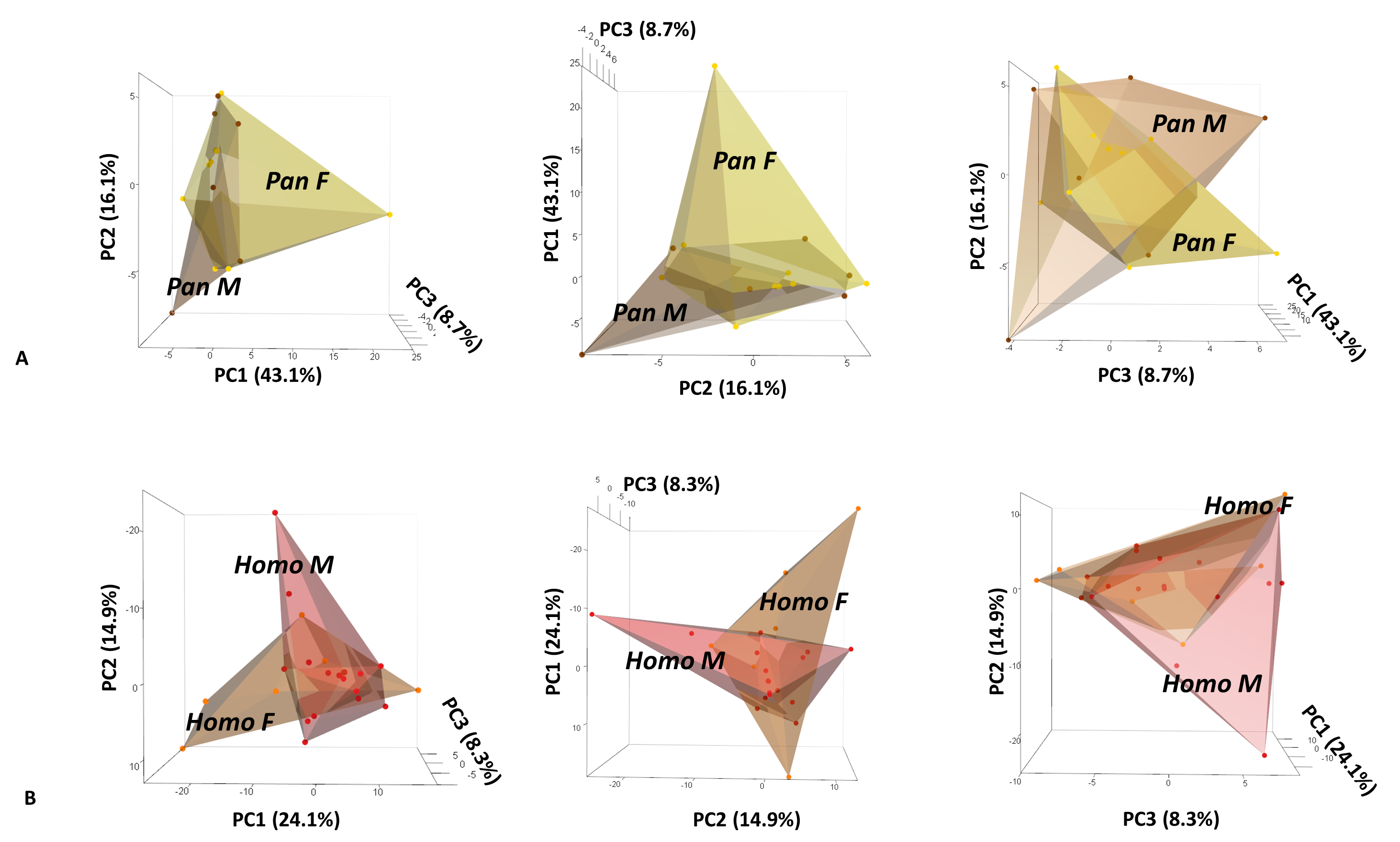

Supplement: Supplementary file 6 — Figure S6 PCA of rBV/TV distribution in the proximal tibia of (A) Pan and (B) Homo showing no separation between sexes. F, female; M, male. [file AJPA-187-e70084-s001.tif]

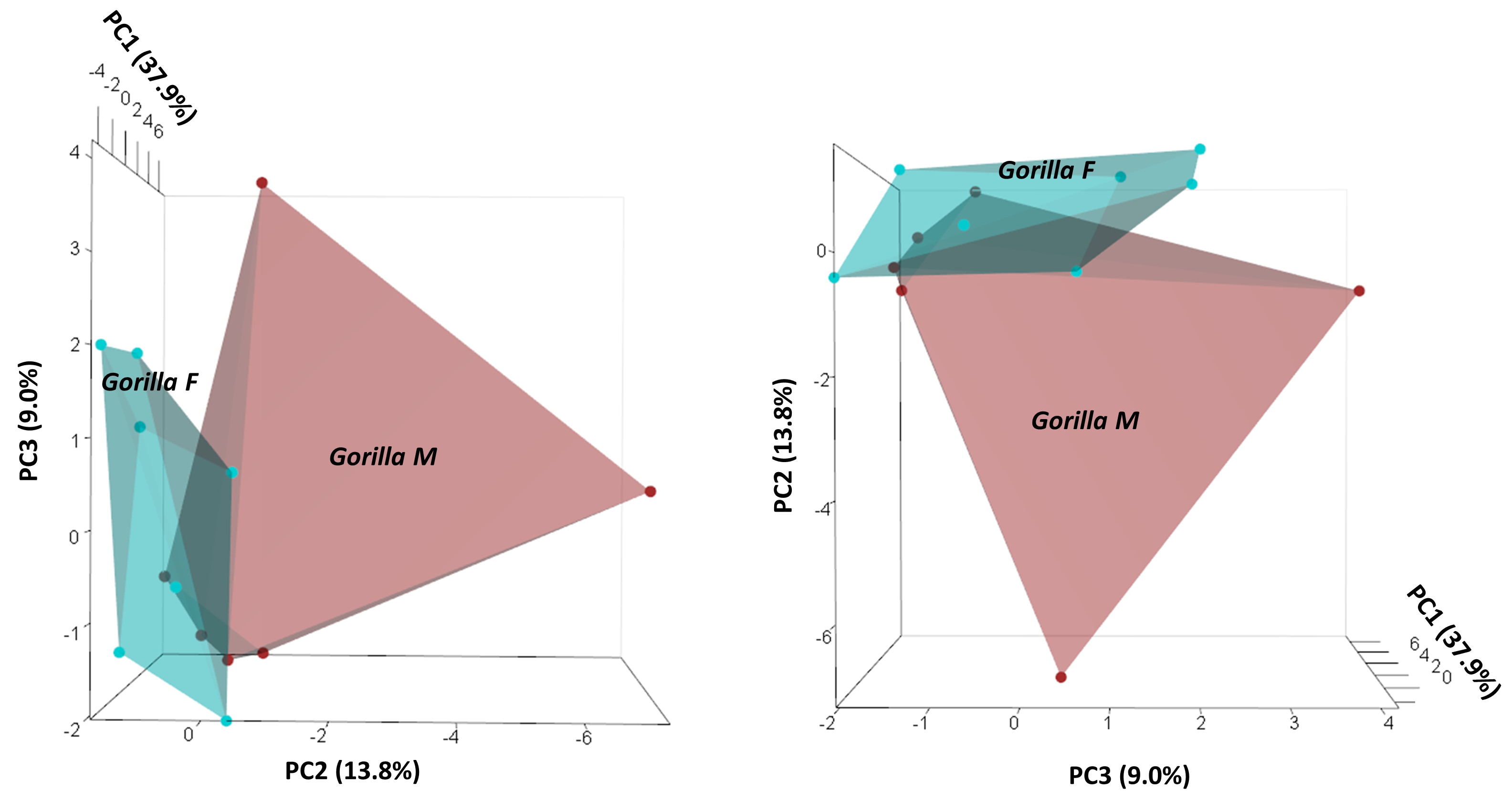

Supplement: Supplementary file 7 — Figure S7 PCA of DA distribution in proximal tibia of Gorilla showing no separation between sexes on PC2 and PC3. F, female; M, male. [file AJPA-187-e70084-s009.tif]

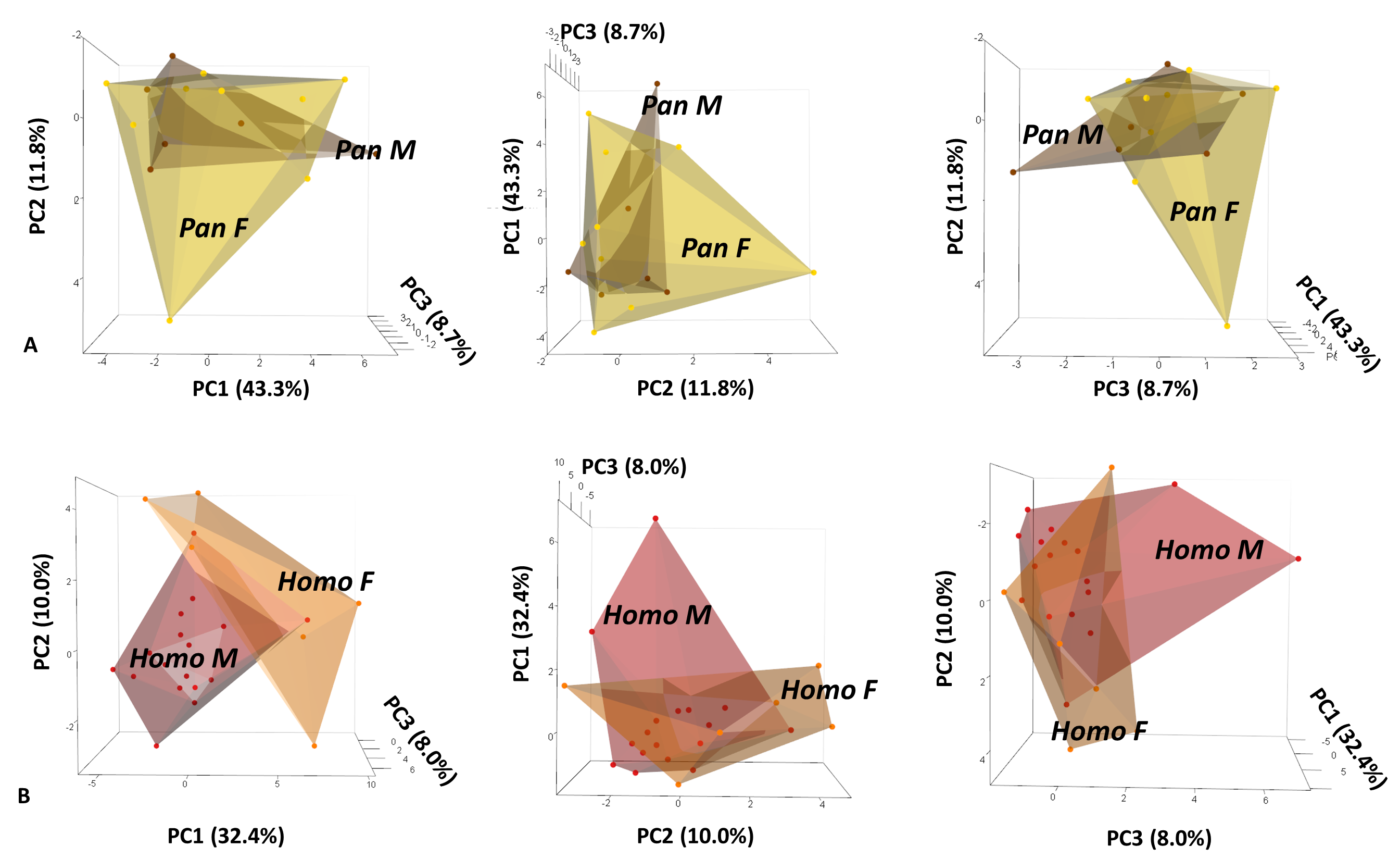

Supplement: Supplementary file 8 — Figure S8 PCA of DA distribution in proximal tibia of (A) Pan, and (B) Homo showing no separation between sexes. F, female; M, male. [file AJPA-187-e70084-s002.tif]
