# Supplementary material for: Trabecular Architecture of the Proximal Tibia in Extant Hominids
Source: Am J Biol Anthropol. 2025 Jun 30;187(3):e70084. doi: 10.1002/ajpa.70084 (PMC12207363; doi:10.1002/ajpa.70084)
Supplement: Supplementary file 9 — Table S1 Complete information of sample composition. Table S2: Descriptive statistics of BV/TV distribution in tibial plateau. [file AJPA-187-e70084-s007.docx]

Supplementary material

| **Table S1:** Complete information of sample composition. | | |  |  |  |  |  |
| --- | --- | --- | --- | --- | --- | --- | --- |
| **Taxonomy** | **Specimen** | **Side** | **Resolution** | **Sex** | **Age** | **Dated** | **Institution** |
| *Homo sapiens* | GAUG-Inden_101 | R | 0.036 | F | adult | 1.0 kya - present | University of Göttingen, Göttingen, Germany |
| *Homo sapiens* | GAUG-Inden_113 | L | 0.036 | M | adult | 1.0 kya - present | University of Göttingen, Göttingen, Germany |
| *Homo sapiens* | GAUG-Inden_117 | L | 0.036 | M | adult | 1.0 kya - present | University of Göttingen, Göttingen, Germany |
| *Homo sapiens* | GAUG-Inden_118 | R | 0.036 | F | adult | 1.0 kya - present | University of Göttingen, Göttingen, Germany |
| *Homo sapiens* | GAUG-Inden_166 | R | 0.036 | M | adult | 1.0 kya - present | University of Göttingen, Göttingen, Germany |
| *Homo sapiens* | GAUG-Inden_243 | R | 0.036 | M | adult | 1.0 kya - present | University of Göttingen, Göttingen, Germany |
| *Homo sapiens* | GAUG-Inden_311 | L | 0.036 | M | adult | 1.0 kya - present | University of Göttingen, Göttingen, Germany |
| *Homo sapiens* | GAUG-Inden_91 | R | 0.036 | M | adult | 1.0 kya - present | University of Göttingen, Göttingen, Germany |
| *Homo sapiens* | GAUG-Inden_93 | R | 0.036 | F | adult | 1.0 kya - present | University of Göttingen, Göttingen, Germany |
| *Homo sapiens* | NGA-88_SK-1047 | R | 0.030 | F | adult | 1.0 kya - present | University of Kent, Canterbury, United Kingdom |
| *Homo sapiens* | NGA-88_SK-766 | R | 0.030 | M | adult | 1.0 kya - present | University of Kent, Canterbury, United Kingdom |
| *Homo sapiens* | NGA-88_SK-825 | L | 0.030 | F | adult | 1.0 kya - present | University of Kent, Canterbury, United Kingdom |
| *Homo sapiens* | NGA-88_SK-880 | L | 0.030 | M | adult | 1.0 kya - present | University of Kent, Canterbury, United Kingdom |
| *Homo sapiens* | NGA-88_SK-911 | L | 0.030 | F | adult | 1.0 kya - present | University of Kent, Canterbury, United Kingdom |
| *Homo sapiens* | NGB-89_SK-15 | R | 0.036 | N/A | adult | 1.0 kya - present | University of Kent, Canterbury, United Kingdom |
| *Homo sapiens* | MRT_FCS73 | R | 0.038 | M | adult | 1.0 kya - present | Mary Rose Trust, Portsmouth, United Kingdom |
| *Homo sapiens* | MRT_FCS47 | L | 0.036 | M | adult | 1.0 kya - present | Mary Rose Trust, Portsmouth, United Kingdom |
| *Homo sapiens* | MRT_FCS26 | L | 0.036 | M | adult | 1.0 kya - present | Mary Rose Trust, Portsmouth, United Kingdom |
| *Homo sapiens* | MRT_FCS79 | R | 0.035 | M | adult | 1.0 kya - present | Mary Rose Trust, Portsmouth, United Kingdom |
| *Homo sapiens* | MRT_FCS35 | L | 0.038 | M | adult | 1.0 kya - present | Mary Rose Trust, Portsmouth, United Kingdom |
| *Homo sapiens* | MRT_FCS50 | L | 0.033 | M | adult | 1.0 kya - present | Mary Rose Trust, Portsmouth, United Kingdom |
| *Homo sapiens* | MRT_FCS8 | L | 0.038 | M | adult | 1.0 kya - present | Mary Rose Trust, Portsmouth, United Kingdom |
| *Homo sapiens* | MRT_FCS11 | L | 0.034 | M | adult | 1.0 kya - present | Mary Rose Trust, Portsmouth, United Kingdom |
| *Homo sapiens* | MRT_FCS40 | L | 0.034 | M | adult | 1.0 kya - present | Mary Rose Trust, Portsmouth, United Kingdom |
| *Homo sapiens* | MRT_FCS6 | R | 0.038 | M | adult | 1.0 kya - present | Mary Rose Trust, Portsmouth, United Kingdom |
| *P. troglodytes verus* | MPITC_15001 | R | 0.030 | F | adult | 1.0 kya - present | Max Planck Institute for Evolutionary Anthropology, Leipzig, Germany |
| *P. troglodytes verus* | MPITC_11778 | R | 0.030 | F | adult | 1.0 kya - present | Max Planck Institute for Evolutionary Anthropology, Leipzig, Germany |
| *P. troglodytes verus* | MPITC_11781 | R | 0.030 | M | adult | 1.0 kya - present | Max Planck Institute for Evolutionary Anthropology, Leipzig, Germany |
| *P. troglodytes verus* | MPITC_11785 | R | 0.030 | M | adult | 1.0 kya - present | Max Planck Institute for Evolutionary Anthropology, Leipzig, Germany |
| *P. troglodytes verus* | MPITC_11786 | R | 0.030 | F | adult | 1.0 kya - present | Max Planck Institute for Evolutionary Anthropology, Leipzig, Germany |
| *P. troglodytes verus* | MPITC_11793 | R | 0.030 | M | adult | 1.0 kya - present | Max Planck Institute for Evolutionary Anthropology, Leipzig, Germany |
| *P. troglodytes verus* | MPITC_15012 | R | 0.030 | M | adult | 1.0 kya - present | Max Planck Institute for Evolutionary Anthropology, Leipzig, Germany |
| *P. troglodytes verus* | MPITC_15013 | R | 0.030 | F | adult | 1.0 kya - present | Max Planck Institute for Evolutionary Anthropology, Leipzig, Germany |
| *P. troglodytes verus* | MPITC_15014 | R | 0.030 | M | adult | 1.0 kya - present | Max Planck Institute for Evolutionary Anthropology, Leipzig, Germany |
| *P. troglodytes verus* | MPITC_15019 | R | 0.030 | M | adult | 1.0 kya - present | Max Planck Institute for Evolutionary Anthropology, Leipzig, Germany |
| *P. troglodytes verus* | MPITC_15023 | R | 0.030 | F | adult | 1.0 kya - present | Max Planck Institute for Evolutionary Anthropology, Leipzig, Germany |
| *P. troglodytes verus* | MPITC_15004 | R | 0.030 | F | adult | 1.0 kya - present | Max Planck Institute for Evolutionary Anthropology, Leipzig, Germany |
| *P. troglodytes verus* | MPITC_11775 | R | 0.030 | F | adult | 1.0 kya - present | Max Planck Institute for Evolutionary Anthropology, Leipzig, Germany |
| *P. troglodytes verus* | MPITC_11800 | R | 0.030 | F | adult | 1.0 kya - present | Max Planck Institute for Evolutionary Anthropology, Leipzig, Germany |
| *P. troglodytes verus* | MPITC_14994 | R | 0.030 | F | adult | 1.0 kya - present | Max Planck Institute for Evolutionary Anthropology, Leipzig, Germany |
| *Gorilla gorilla gorilla* | PC_CAMI_106 | L | 0.050 | M | adult | 1.0 kya - present | Powell-Cotton Museum, Birchington-on-Sea, United Kingdom |
| *Gorilla gorilla gorilla* | PC_FC_123 | L | 0.063 | M | adult | 1.0 kya - present | Powell-Cotton Museum, Birchington-on-Sea, United Kingdom |
| *Gorilla gorilla gorilla* | PC_MER_329 | L | 0.058 | F | adult | 1.0 kya - present | Powell-Cotton Museum, Birchington-on-Sea, United Kingdom |
| *Gorilla gorilla gorilla* | PC_MER_720 | R | 0.056 | M | adult | 1.0 kya - present | Powell-Cotton Museum, Birchington-on-Sea, United Kingdom |
| *Gorilla gorilla gorilla* | PC_MER_840 | L | 0.055 | F | adult | 1.0 kya - present | Powell-Cotton Museum, Birchington-on-Sea, United Kingdom |
| *Gorilla gorilla gorilla* | PC_MER_856 | R | 0.034 | F | adult | 1.0 kya - present | Powell-Cotton Museum, Birchington-on-Sea, United Kingdom |
| *Gorilla gorilla gorilla* | PC_MER_798 | L | 0.034 | F | adult | 1.0 kya - present | Powell-Cotton Museum, Birchington-on-Sea, United Kingdom |
| *Gorilla gorilla gorilla* | PC_MER_372 | R | 0.043 | M | adult | 1.0 kya - present | Powell-Cotton Museum, Birchington-on-Sea, United Kingdom |
| *Gorilla gorilla gorilla* | PC_MER_300 | R | 0.043 | F | adult | 1.0 kya - present | Powell-Cotton Museum, Birchington-on-Sea, United Kingdom |
| *Gorilla gorilla gorilla* | PC_MER_135 | R | 0.045 | M | adult | 1.0 kya - present | Powell-Cotton Museum, Birchington-on-Sea, United Kingdom |
| *Gorilla gorilla gorilla* | PC_MER_96 | L | 0.040 | F | adult | 1.0 kya - present | Powell-Cotton Museum, Birchington-on-Sea, United Kingdom |
| *Gorilla gorilla gorilla* | PC_M36_264 | R | 0.050 | M | adult | 1.0 kya - present | Powell-Cotton Museum, Birchington-on-Sea, United Kingdom |
| *Gorilla gorilla gorilla* | PC_MER_95 | L | 0.038 | F | adult | 1.0 kya - present | Powell-Cotton Museum, Birchington-on-Sea, United Kingdom |
| *Pongo pygmaeus* | ZMB_MAN_83213 | L | 0.029 | M | adult | 1.0 kya - present | Natural History Museum, Berlin, Germany |
| *Pongo sp.* | ZSM_1982_0092 | R | 0.030 | F | adult | 1.0 kya - present | Bavarian State Collection of Zoology, Munich, Germany |
| *Pongo pygmaeus pygmaeus* | ZSM_1907_0633b | R | 0.025 | F | adult | 1.0 kya - present | Bavarian State Collection of Zoology, Munich, Germany |
| *Pongo pygmaeus pygmaeus* | ZSM_1907_0489 | L | 0.028 | F | adult | 1.0 kya - present | Bavarian State Collection of Zoology, Munich, Germany |
| *Pongo pygmaeus* | ZSM_1966_0203 | L | 0.030 | M | adult | 1.0 kya - present | Bavarian State Collection of Zoology, Munich, Germany |
| *Pongo abelii* | ZSM_1973_0270 | R | 0.025 | F | adult | 1.0 kya - present | Bavarian State Collection of Zoology, Munich, Germany |
| *Pongo pygmaeus pygmaeus* | ZSM_1909_0801 | R | 0.030 | F | adult | 1.0 kya - present | Bavarian State Collection of Zoology, Munich, Germany |

| **Table S2:** Descriptive statistics of BV/TV distribution in tibial plateau. | | | | | |  |  |
| --- | --- | --- | --- | --- | --- | --- | --- |
| **Taxa** | **Region** | **n** | **mean** | **SD** | **min** | **max** | **median** |
| *Homo* | tibial plateau | 25 | 0.237 | 0.056 | 0.147 | 0.353 | 0.236 |
|  | lateral |  | 0.235 | 0.061 | 0.135 | 0.359 | 0.234 |
|  | medial |  | 0.224 | 0.050 | 0.148 | 0.338 | 0.228 |
| *Gorilla* | tibial plateau | 13 | 0.317 | 0.030 | 0.258 | 0.352 | 0.323 |
|  | lateral |  | 0.305 | 0.028 | 0.249 | 0.337 | 0.309 |
|  | medial |  | 0.315 | 0.032 | 0.254 | 0.363 | 0.322 |
| *Pan* | tibial plateau | 15 | 0.317 | 0.051 | 0.239 | 0.402 | 0.311 |
|  | lateral |  | 0.310 | 0.053 | 0.215 | 0.302 | 0.397 |
|  | medial |  | 0.311 | 0.050 | 0.236 | 0.401 | 0.311 |
| *Pongo* | tibial plateau | 7 | 0.239 | 0.029 | 0.192 | 0.281 | 0.237 |
|  | lateral |  | 0.225 | 0.032 | 0.176 | 0.261 | 0.225 |
|  | medial |  | 0.243 | 0.027 | 0.198 | 0.288 | 0.246 |

**Figure S1:** Standard deviation maps of rBV/TV values in the proximal tibia of *Homo, Gorilla, Pan,* and *Pongo.* Vertical and horizontal lines through the SD models show where the cross-sectional sagittal and coronal planes are positioned. Red colour shows the highest variability in the rBV/TV values and blue colour shows the lowest variability in the rBV/TV values. L, lateral; M, medial.

**Figure S2:** PC3 of rBV/TV distribution in proximal femur of *Homo*, *Gorilla*, *Pan*, and *Pongo* showing separation among studied taxa. Models at the end of each axis represent the regions of high rBV/TV driving variance along PC3. Models demonstrate the rBV/TV values separating between *Pongo* (positive PC3+3SD) and *Pan* (negative PC3-3SD). L, lateral; M, medial.

**Figure S3:** Standard deviation maps of DA distribution of the proximal tibia of *Homo, Gorilla, Pan,* and *Pongo.* Red colour shows the highest variability in the DA values and blue colour shows the lowest variability in the DA values. L, lateral; M, medial.

**Figure S4:** PCA of DA distribution in the proximal tibia of *Homo*, *Gorilla*, *Pan*, and *Pongo* on PC3.

**Figure S5:** *Gorilla* mean models and PCA of rBV/TV distribution in the proximal tibia of *Gorilla* showing no separation on PC1 and partial separation on PC2 and PC3. Horizontal lines through the superior view mean models show where the cross-sectional coronal planes are positioned. Circles in the superior and anterior views represent the homologous locations. F, female; M, male.

**Figure S6:** PCA of rBV/TV distribution in the proximal tibia of (A) *Pan* and (B) *Homo* showing no separation between sexes. F, female; M, male.

**Figure S7:** PCA of DA distribution in proximal tibia of *Gorilla* showing no separation between sexes on PC2 and PC3. F, female; M, male.

**Figure S8:** PCA of DA distribution in proximal tibia of (A) *Pan,* and (B) *Homo* showing no separation between sexes. F, female; M, male.
